# Contrasting effects of shooting disturbance on the movement and behavior of sympatric wildfowl species

**DOI:** 10.1002/eap.3032

**Published:** 2024-10-25

**Authors:** Luke Ozsanlav‐Harris, Aimée L. S. McIntosh, Larry R. Griffin, Geoff M. Hilton, Lei Cao, Jessica M. Shaw, Stuart Bearhop

**Affiliations:** ^1^ Centre for Ecology and Conservation University of Exeter, Penryn Cornwall UK; ^2^ Wildfowl & Wetlands Trust, Slimbridge Gloucester UK; ^3^ NatureScot Stilligarry Isle of South Uist UK; ^4^ ECO‐LG Ltd Dumfries UK; ^5^ State Key Laboratory of Urban and Regional Ecology, Research Center for Eco‐Environmental Sciences Chinese Academy of Sciences Beijing China

**Keywords:** accelerometer, barnacle goose, biologging, habitat selection, human−wildlife conflict, hunting, landscape of fear, movement, white‐fronted goose

## Abstract

Human−wildlife conflict is a global conservation issue, necessitating effective mitigation strategies. Hunting is a common management approach to reduce conflict, but the indirect consequences are often overlooked. Chronic hunting‐related disturbance can reduce fitness and redistribute species. In recent decades, goose−agricultural conflict has intensified due to increasing abundance and shifts towards agricultural foraging. On Islay, Scotland, escalating conflict culminated in shooting Greenland barnacle geese *Branta leucopsis* to reduce damage to agricultural grassland. In this study, we contrast the impact of shooting disturbance on the movement, behavior, energy expenditure and habitat selection of the target species (Greenland barnacle goose) and a vulnerable nontarget species (Greenland white‐fronted goose, *Anser albifrons flavirostris*) using biologging devices (target species: *n* = 33; nontarget species: *n* = 94). Both species were displaced by shooting, and greater distances were subsequently traveled by the target species (1.71 km when directly targeted). When disturbed at any distance, total daily movement increased significantly by 1.18 km for the target species but not for the nontarget species. The target species exhibited no accompanying change in diurnal energy expenditure (measured via accelerometery) but foraged in improved grasslands further from roads after shooting disturbance, where disturbance from all sources was likely lower. The significant increases in movement and changes in foraging site selection of the target species could reduce fitness but given the infrequency of shooting disturbances (0.09 per day) there is likely capacity for compensatory feeding to recoup energetic losses. The nontarget species expectedly showed no significant change in energy expenditure, behavior or habitat selection following shooting disturbance, suggesting mitigation strategies have been effective at minimizing fitness impacts. Refuge areas with a 3.5 km diameter (three times the maximum distance from shooting that displacement was detectable) could provide undisturbed foraging for the target species, minimizing compensatory feeding and further agricultural damage. Wildlife managers should, where possible, consider the fitness implications of shooting disturbance, and whether compensatory feeding and redistribution could hamper conflict mitigation. Management strategies should also include species‐specific monitoring and mitigation as we have demonstrated differing responses potentially due to imposed mitigation but also differing species ecology and “landscapes of fear.”

## INTRODUCTION

Human−wildlife conflict is an acute global conservation issue. The ongoing encroachment of humans into the natural environment and the often conflicting needs of wildlife conservation and people only exacerbates conflict (Redpath et al., 2013). Unmitigated conflict can have severe social and economic ramifications for human communities (Barua et al., 2013; Mackenzie & Ahabyona, 2012), which can subsequently reduce local support for conservation and lead to retaliatory killing of conflict‐causing species (Mateo‐Tomás et al., 2012; McManus et al., 2015). The implementation of effective and sustainable mitigation strategies that balance anthropogenic needs with conservation objectives are vital for reducing conflict and encouraging coexistence (Dickman, 2010; Madden & McQuinn, 2014; Nyhus, 2016).

Management strategies to reduce conflict include lethal control, scaring (King et al., 2017), translocation (Massei et al., 2010), and compensatory payments (McManus et al., 2015). These strategies generally focus on the populations that cause conflict, but often affect other species within the management environment (Mustin et al., 2018; Norvell et al., 2014). For example, poison‐baiting for invasive feral cats and foxes in Australia and New Zealand killed native nontarget species (Dundas et al., 2014; Glen et al., 2007). While human−wildlife coexistence approaches have gained favor in recent years, removing wildlife through lethal control, that is, hunting and culling, remains key to many management strategies (Treves & Ullas Karanth, 2003; van der Jeugd & Kwak, 2017). These control schemes mitigate conflict by reducing the population engaged in conflict‐inducing behavior (Geisser & Reyer, 2004) or by removing specific problem individuals (Swan et al., 2017). Impact assessments of shooting management typically focus on the impact of mortality on population dynamics, such as productivity and abundance, to prevent overexploitation (Gamelon et al., 2021; Johnson et al., 2018; Koons et al., 2014). However, shooting has further implications, such as altering movement (Thorsen et al., 2022), time‐activity budgets, and resource use (Atkins et al., 2019; Crosmary et al., 2012).

The “landscape of fear” describes the spatiotemporal variability in an animal's behavior and habitat selection in response to the perceived pattern of predation risk (Laundré et al., 2001, 2010). Shooting can act as a proxy for natural predation thereby altering the fear landscape directly through mortality and indirectly through disturbance (Gaynor et al., 2019; Månsson, 2017). By changing the perceived predation risk, shooting disturbance stimulates anti‐predation behavior such as increased movement (Kloppers et al., 2005), vigilance (Casas et al., 2009; Li et al., 2010), and stress hormone secretion (Atkins et al., 2017). Furthermore, shooting disturbance can cause temporally and energetically costly redistribution and site avoidance, forcing disturbed individuals to forage on low‐quality habitat (Dwinnell et al., 2019; Michel et al., 2016). This can increase immediate mortality risk, due to starvation, and carry over effects on long‐term fitness such as reproductive success and migratory performance (Bauer et al., 2018; Béchet et al., 2004). Compensatory behaviors including increasing intake rates (Nolet et al., 2016), delaying migration departure (Bauer et al., 2018; Béchet et al., 2004), and redistributing to alternative sites can offset energetic losses, but risk exacerbating conflict (Olson et al., 2015; Santiago‐Avila et al., 2018).

Shooting management can be nonspecific in nature and can negatively impact nontarget species. Direct mortality and sub‐lethal wounding of nontarget species can be monitored and reduced through management controls such as regulation of hunting season (Madsen et al., 2016) and educating hunters in species identification (Clausen et al., 2017; Noer et al., 2007). However, behavioral responses of nontarget species to shooting disturbance can be challenging to identify (Little et al., 2016; Stankowich, 2008) and are often overlooked during the development and assessment of management plans (Cromsigt et al., 2013). Furthermore, risk perception can differ among species and very little attention has been given to the behavioral response of nontarget species to disturbance (Grignolio et al., 2011; Mori, 2017; Zaccaroni et al., 2012). Where nontarget species are of conservation concern, additional fitness costs of disturbance could increase declines of already vulnerable populations and put management objectives at odds with those of conservation, risking further antagonism among stakeholders.

Recent increases of many European goose populations have resulted in conflicts with agricultural stakeholders, because of their preference for agricultural grassland coupled with human efforts to intensify pasture management and increase stock density (Bjerke et al., 2021; Montràs‐Janer et al., 2020). Schemes aimed at reducing goose−agriculture conflict typically include compensatory payments and scaring, but shooting management to reduce populations and redistribute individuals away from conflict sites is also used (Cope et al., 2006; Klok et al., 2010; Williams et al., 2019). The island of Islay, off the west coast of Scotland, is an important wintering site for several goose species, and has a history of goose−agriculture conflict (Mckenzie & Shaw, 2017). The nonbreeding population of Greenland barnacle geese (*Branta leucopsis*, GBG hereafter) on the island increased from ~3000 in the 1950s to ~50,000 in the early 2000s due to improved grassland expansion and conservation measures to control hunting (Mason et al., 2017). Consequently, conflict among stakeholders (farmers, conservation groups, and government organizations) has increased in recent years (Mckenzie & Shaw, 2017).

Since the early 2000s, shooting of GBG was used as lethal scaring, and from 2014, population control was introduced to reduce damage to agricultural grassland on Islay (Mckenzie, 2014; Mckenzie & Shaw, 2017). Population control was part of the Islay Sustainable Goose Management Strategy 2014 (ISGMS), implemented by NatureScot (public body responsible for Scotland's natural heritage) to reduce the Islay nonbreeding population of GBG by up to 25%–30%, largely using shooting by licensed marksmen on agricultural grassland (Mckenzie, 2014). The ISGMS monitors the abundance and harvest of GBG to ensure long‐term population viability because GBG are an Annex 1 protected species under the European Union Birds Directive. However, the indirect fitness costs associated with behavioral and movement responses to shooting disturbance are not yet quantified. Additionally, in winter on Islay, the GBG live in sympatry with the Greenland white‐fronted goose (*Anser albifrons flavirostris*, GWfG hereafter), an “Endangered” sub‐species under International Union for Conservation of Nature Red‐list criteria (Stroud et al., 2012). The Islay nonbreeding GWfG population declined from ~13,500 in 1999 to 5297 birds in 2021 (Fox et al., 2022), likely due to poor breeding success (Ozsanlav‐Harris et al., 2023). As a result, they are not targeted during shooting. However, as they forage in sympatry with GBG it is important to assess the impacts of shooting disturbance on GWfG, although marksmen are instructed not to shoot at mixed flocks of GBG and GWfG. Given the status of this population, any additional fitness costs because of the disturbance could jeopardize recovery (Griffin et al., 2020).

**FIGURE 1:**
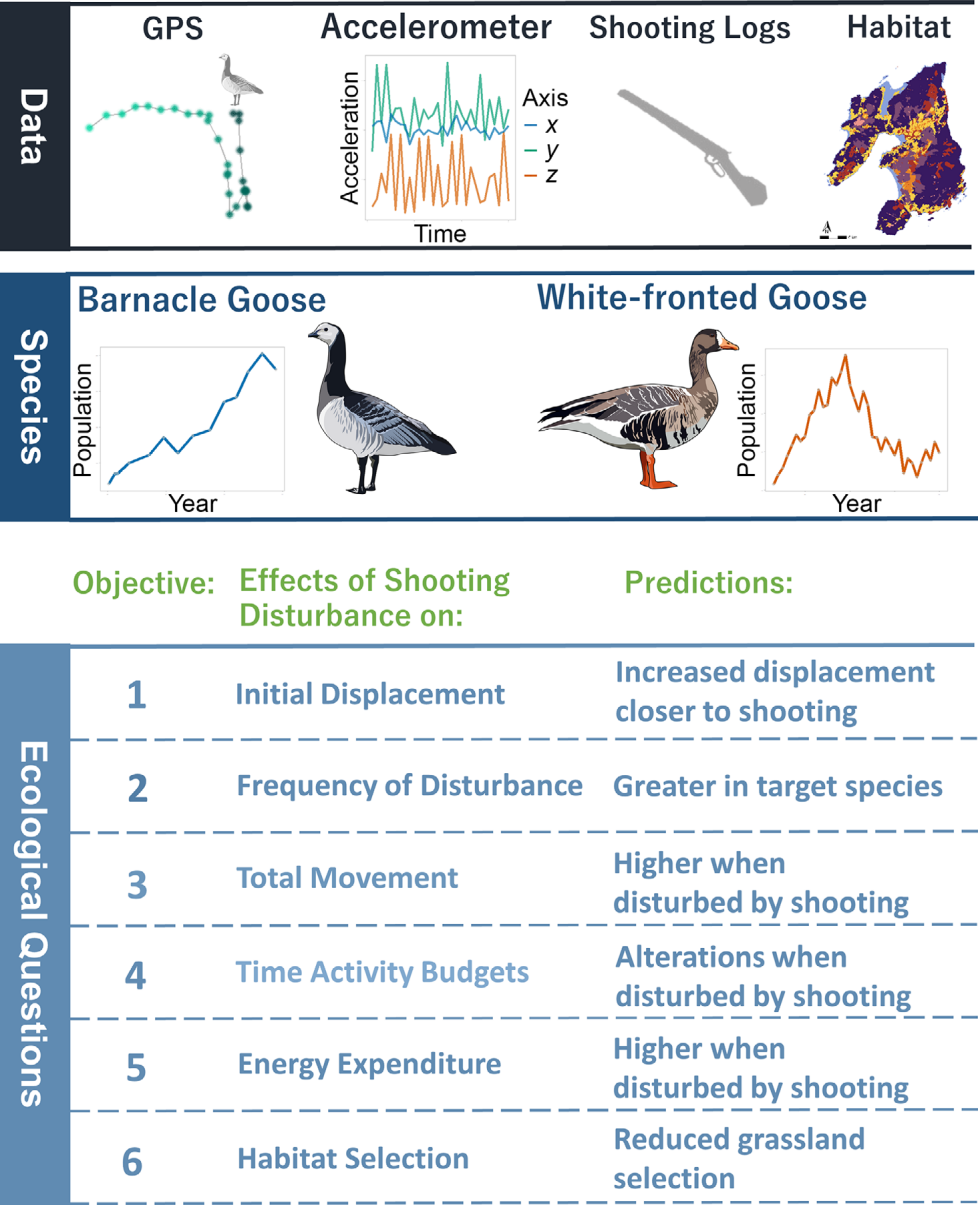
Overview of multiple analysis testing for the effects of shooting disturbance on the movement, behavior, and energy expenditure of nonbreeding geese on Islay, Scotland. Illustration copyright owned by Luke Ozsanlav‐Harris (GWfG) and Aimée L. S. McIntosh (GBG).

This system offers a unique opportunity to assess the impact of shooting disturbance across the nonbreeding period on both target and nontarget species by combining biologging data with shooting records at fine spatiotemporal resolution. Here, we assess the impacts of shooting disturbance on movement (predictions 1 and 3), time activity budgets (prediction 4), energy expenditure (prediction 5) and habitat use (prediction 6), as well as quantifying the frequency that individuals are disturbed (prediction 2, see Figure 1 for an overview). We make the following predictions:

1. Prediction 1: Shooting disturbance will cause flight initiation but this effect will decay with increasing distance from the shooting event.
2. Prediction 2: The target species (GBG) will be disturbed at a greater frequency than the nontarget species (GWfG).
3. Prediction 3: Daily movement distances will be greater on days individuals are exposed to shooting.
4. Prediction 4: Shooting disturbance will increase total flight duration as individuals are displaced, increase compensatory feeding and increase vigilance due to heightened sensitivity to post‐shooting stimuli.
5. Prediction 5: Energy expenditure (measured using accelerometers) will be greater on days individuals are exposed to shooting due to increased flight and compensatory feeding.
6. Prediction 6: Individuals disturbed by shooting will show weaker selection for preferred improved grassland and/or select for sites further from shooting activity and forms of anthropogenic disturbance, namely, further from roads (Jensen et al., 2016; Simonsen et al., 2016).

Generally, we predict that there is an energetic cost of shooting disturbance in both species but the costs for the nontarget species are diminshed.

## METHODS

### Study site and species

**FIGURE 2:**
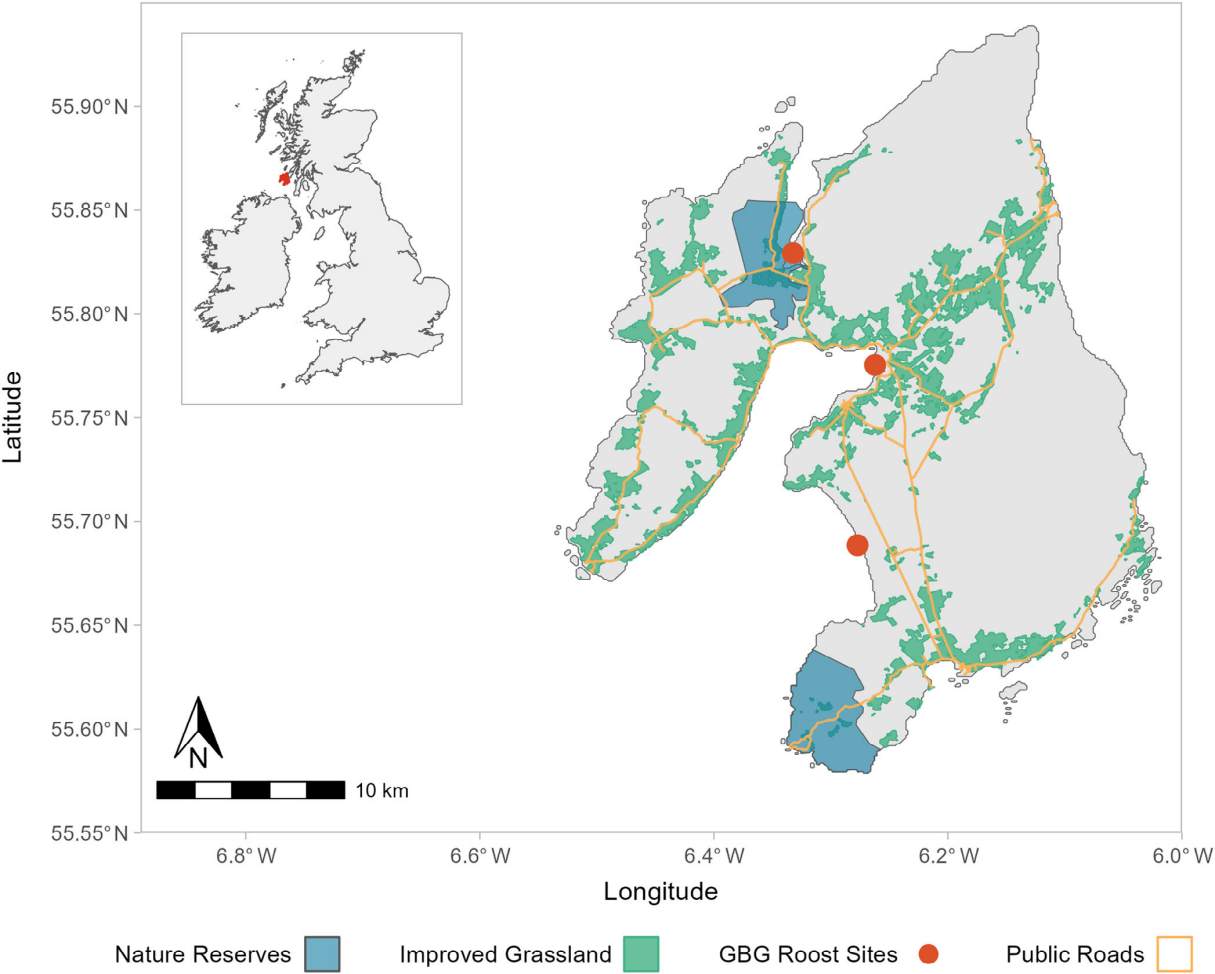
Map of Islay, Scotland. Depicted is the public road network, the three largest Greenland barnacle geese (GBG) roost sites, and the two largest nature reserves on the island. White‐fronted goose roosts are not included as they are numerous, small, and transient. Shooting does not take place within nature reserves or around any roost sites.

GBG and GWfG spend the nonbreeding period in the northern and western regions of Scotland and Ireland, arriving in October to November and departing in April. Islay is an island (62,000 ha) on the west coast of Scotland (Figure 2). Both goose taxa preferentially utilize improved grassland on Islay, especially recently reseeded pasture, resulting in highly overlapping foraging ranges. Shooting of GBG is permitted according to Article 9 of the Birds Directive to prevent serious agricultural damage and is only permitted on approximately 15%–20% of land within the Islay Local Goose Management Scheme (which implements the ISGMS; Mckenzie & Shaw, 2017). Shooting is coordinated by NatureScot and is predominantly conducted by licensed marksmen (>80%) between November and April but is spatially restricted to first‐ and second‐year reseeded fields identified at the start of the shooting season. Flocks of GBG are shot by licensed marksmen or farmers opportunistically throughout the winter when foraging on permitted fields, although not when GBG are foraging in mixed flocks with GWfG.

### GPS tracking

Geese were caught at sites across Islay to include birds utilizing different foraging and roosting sites (full details in Appendix S1: Section S1). GBG were captured in winters 2019/2020 and 2020/2021 and three types of solar‐powered neck collar tags were deployed: nanoFix GEO + RF (Pathrack Ltd, Otley, UK, 15g, *n* = 13), Ecotone GPS‐GSM (Ecotone, Gydnia, Poland, 25g, *n* = 9), and Ornitela OT‐NL40 GPS‐GSM (Ornitela, Vilnius, Lithuania, 19g, *n* = 16). GWfG were captured every winter from 2013/2014 until 2020/2021 and three types of solar‐powered neck collar tags were deployed: Ornitela model N38 GPS‐GSM (Ornitela, Vilnius, Lithuania, c37g, *n* = 24), Ecotone GPS‐GSM (Ecotone, Gydnia, Poland, c24g, *n* = 41), and Ecotone GPS‐UHF (Ecotone, Gydnia, Poland, c24g, *n* = 29). GPS data were obtained from all tags and triaxial accelerometery data from Ecotone and Ornitela GSM tags only (see Appendix S1 for sampling schedule). All GPS data were re‐sampled to 1‐h intervals prior to analysis to ensure the straight‐line distance between fixes was comparable across individuals (Noonan et al., 2019).

### Shooting logs

Detailed records of shooting by marksmen have been maintained by NatureScot since shooting began in the early 2000s. These records contain the date, time, number of shots fired, number of dead birds recovered, and the exact land parcel where each shooting event took place (regardless of whether it resulted in fatalities).

### General statistical procedures

All generalized linear mixed‐effect models were fit in R v3.6 using the package *glmmTMB* (Brooks et al., 2017). Models were checked for violations of the underlying distributional assumptions using the *DHARMa* (Hartig, 2021) and *Performance* packages (Lüdecke et al., 2021). To compare models, Akaike information criterion (AIC) was calculated, unless stated, and the model with the lowest AIC chosen as the most parsimonious model, regardless of the number of parameters. Many of the models include response variables that are successive daily measurements and therefore form time‐series, for example travel distances, time‐activity budgets and overall dynamic body acceleration (ODBA). These daily measurements are nonindependent due to temporal autocorrelation. Therefore, any time series data were fit with a first‐order autoregressive covariance structure (AR1). This covariance structure assumes data from consecutive days or nights for an individual are more strongly correlated than those at greater time‐lags. For daily measurements using accelerometer data (activity‐time budget and ODBA analysis), days/nights with less than six accelerometer bursts were removed from analysis.

Several predictor variables are included in multiple models. We describe these here in detail and then give a brief account where they occur subsequently.

1. Species (*Sp*): A two‐level categorical variable indicating the species (GBG or GWfG).
2. Sex (*Sex*): A two‐level categorical variable indicating the sex of a bird (male or female).
3. Time of year (*Date*): A continuous variable indicating the integer number of days since the 1 November within the same winter period (1 November 2021 = 1; 2 January 2022 = 63).
4. Winter (*Win*): A categorical variable where each level runs from October in one year to April in the subsequent year, for example October 2021–April 2022. The number of levels in *Win* differs between datasets but is modeled as a random effect in all models unless this leads to model convergence issues (likely due to insufficient levels in the variable).

### Prediction 1: Displacement due to shooting

We tested whether birds were displaced by shooting and how the effect decayed with increasing distance from the shooting event. For each individual, the GPS fix immediately prior to each shooting event was identified and subsequently retained if the fix was recorded within 1 h and 4 km of the shooting event (calculated as the distance between the GPS fix and the centroid of the field where shooting occurred). The period between a retained fix and the following fix chronologically is when an individual could be exposed to shooting disturbance and is called time‐step, *t*, and has an associated step‐length, *SL*_*t*_. To discern whether *SL*_*t*_ was longer or shorter than expected we compare it with the undisturbed step‐length immediately prior to time‐step *t*, namely, *SL*_*t*−1_, thereby individuals become controls of themselves. We then modeled the difference in step‐lengths between time‐step *t* and time‐step *t* − 1 (*SL*_*t*_ − *SL*_*t*−‐1_). Positive distances indicate the bird moved further during disturbed time‐step *t* compared with time‐step *t* − 1. We used a linear mixed‐effects model to explain variation in the difference between step‐lengths, *SL* (Equation 1). In this model, the log of the distance between the location of the bird at time *t* and the shooting event is included as a fixed effect so that the influence of shooting on displacement can decay as the bird becomes further away from the shooting event. The two species are modeled separately but the global model for each species includes the following fixed effects: (1) log of the continuous distance between the shooting event and the location of the bird at time *t (Dist)*; (2) time of the shooting event as a quadratic term (*Time*), represented as the continuous number of hours since midnight; and (3) winter as a two‐level category in the GBG model only (*Win*). We also fit individual ID (*i*) as random intercept term to account for differences between individuals. The full model for GBG took the following form:

(1) $${\mathrm{SL}}_{\mathrm{iw}}=\alpha +\beta_{1}\log\left({\text{Dist}}_{\mathrm{iw}}\right)+\beta_{2}{\text{Time}}_{\mathrm{iw}}+\beta_{3}{\left({\text{Time}}_{\mathrm{iw}}\right)}^{2}+\beta_{4}{\mathrm{Win}}_{w}+\varepsilon_{i},\varepsilon_{i}\sim N\left(0,\sigma^{2}\right),$$

where ${\mathrm{SL}}_{\mathrm{iw}}$ is the difference in step‐lengths for individual i, in winter w; α is the intercept; the β's are the coefficients for the fixed effect; and $\varepsilon_{i}$ denotes the random effect accounting for differences between individuals. For GWfG, the winter fixed effect is removed and included as a random intercept term, $\varepsilon_{\mathrm{iw}}$. This is because fitting the categorical winter variable as a random effect in the GBG model caused convergence issues due to their only being two levels.

### Prediction 2: Classification of shooting disturbance

**FIGURE 3:**
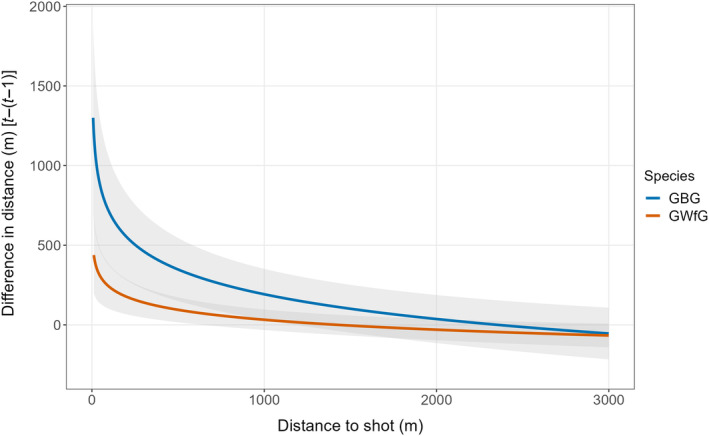
Difference in distance traveled between the inter‐fix period when an individual was exposed to shooting (*t*) and the inter‐fix period immediately prior to shooting (*t* − 1). This was modelled in relation to the distance between the individual and the field that the shooting event took place. Positive distances indicate individuals traveled further when exposed to shooting as opposed to the control period. This relationship was modeled using linear regression analysis with the natural logarithm of the distance to shooting event modeled as a predictor variable. Thin colored lines represent the mean regression line, and the shaded gray area the 95% CI around this mean. GBG, Greenland barnacle geese; GWfG, Greenland white‐fronted goose.

An individual goose on a particular day was “shooting disturbed” if its recorded GPS fixes indicated that it was in close spatiotemporal proximity to a shooting event. A GPS fix was temporally close to a shooting event if it was less than 1 h before a shooting event and in close spatial proximity if it fell within a certain buffer around the centroid of the field that the shooting event took place; 1184 m for GBG and 644 m for GWfG. We used these distances as they were the points at which the lower 95% CI for the relationship between step‐length difference and distance to shooting event (Figure 3) first crossed the line *y* = 0, that is, the first point at which we had less than 95% certainty that the regression line's *y*‐intercept was not 0. Days when an individual was in close spatiotemporal proximity to at least one shooting event were classified as “shooting disturbed” while all others days were classified as “undisturbed.”

### Prediction 3: Differences in daily foraging distance

A minimum of eight fixes per day were required to calculate daytime foraging distance. Cumulative point‐to‐point distance was used to calculate total daily foraging distance traveled (in kilometers) during each goose‐day. This provided a total of 5117 goose days for analysis from 2012 to 2020 (GBG were only tracked from 2019). Each goose‐day was classified as “shooting disturbed” or “undisturbed” as above. To account for spatial autocorrelation in distance traveled values due to geese utilizing fields within the same area, the most frequently visited farm (modal farm ID, *f*) was identified as the farm with the highest number of GPS fixes for each goose‐day. To test for the effect of shooting disturbance on daily distance traveled (*TD*) we fit a generalized linear mixed‐effects model with a gamma error structure (log link function) (Equation 2). The global model was fitted with a first‐order autoregressive covariance structure (AR1) and included the following fixed effects: (1) Shooting disturbance (*Shot*) as a two‐ or three‐level factor (see below); (2) Species (*Sp*); (3) cumulative individual experience of shooting (*Exp*) calculated as the scaled cumulative number days an individual was exposed to shooting within a winter; (4) Sex (*Sex*); and (5) the 3‐way interaction between Disturbance (*Shot*), Species (*Sp*) and Experience (*Exp*). The following random intercept terms were also included: (1) Individual ID (*i*); (2) winter (*w*); and (3) modal farm ID (*f*) to account for pseudo‐replication of individuals within a winter and site fidelity in foraging site selection. The full model took the following form:

(2) $${\mathrm{TD}}_{\text{iwgf}}\sim \text{Gamma}\left(\mu_{\text{iwgf}},\gamma_{\text{iwgf}}\right),\log\left(\mu_{\text{iwgf}}\right)=\alpha +\beta_{1}{\text{Shot}}_{\text{iwgf}}+\beta_{2}{\mathrm{Sp}}_{g}+\beta_{3}{\mathrm{Exp}}_{\text{iwgf}}+\beta_{4}\left({\text{Shot}}_{\text{iwgf}}*{\mathrm{Sp}}_{g}\right)+\beta_{5}\left({\mathrm{Sp}}_{g}*{\mathrm{Exp}}_{\text{iwgf}}\right)+\beta_{6}\left({\text{Shot}}_{\text{iwfg}}*{\mathrm{Sp}}_{g}*{\mathrm{Exp}}_{\text{iwfg}}\right)+\beta_{7}{\mathrm{Sex}}_{\text{iwgf}}+\varepsilon_{\mathrm{iwf}},\varepsilon_{\mathrm{iwf}}\sim N\left(0,\sigma^{2}\right),$$

where *TD*_*iwgf*_ is the total daily distance traveled for individual *i*, in winter *w*, for goose species *g*, at modal farm, *f*; α is the intercept; the β's are the coefficients of fixed effects; and $\varepsilon_{\text{iwgf}}$ denotes crossed random effects accounting for individual *i*, winter *w*, and modal farm, *f*. The shooting disturbance variable (*Shot*) was modeled as either a two‐level factor (shooting disturbed and undisturbed) or a three‐level factor where “shooting disturbed” was divided into within‐field and out‐of‐field disturbance to assess the differences in response to shooting when individuals were in the same field as the shooting event. Species and sex were included in all models to control for differences in foraging distances between these classes. We compared the global model with an a priori model set of seven other models (Table 1) removing the interaction terms with disturbance and experience, and finally the disturbance and experience terms entirely.

**TABLE 1:** Model selection table to understand the immediate and cumulative effects of shooting disturbance on the daily foraging distance of Greenland white‐fronted goose (GWfG) and Greenland barnacle geese (GBG).

*Sp*	*Sex*	*Shot*		*Exp*	Shot*Sp	Shot*Exp	Sp*Exp	Shot*Sp*Exp	*k*	AIC	∆AIC
		*2 levels*	*3 levels*								
X	X	X	…	X	X	X	X	X	15	19,100.4	0.0
X	X	…	X	X	X	X	X	X	19	19,101.2	0.8
X	X	…	X	X	X	…	…	…	14	19,101.4	1.0
X	X	…	X	X	X	…	X	…	15	19,102.2	1.9
X	X	X	…	X	X	…	…	…	12	19,103.4	3.0
X	X	…	X	X	X	X	…	…	16	19,103.7	3.3
X	X	X	…	X	X	…	X	…	13	19,104.3	3.9
X	X	…	X	X	…	…	…	…	12	19,104.4	4.0
X	X	X	…	X	X	X	…	…	13	19,104.4	4.0
X	X	…	X	X	X	X	X	…	17	19,104.7	4.3
X	X	…	X	X	…	…	X	…	13	19,105.0	4.6
X	X	X	…	X	…	…	…	…	11	19,105.0	4.7
X	X	X	…	X	…	X	…	…	12	19,105.1	4.8
X	X	…	X	X	…	X	…	…	14	19,105.3	4.9
X	X	X	…	X	X	X	X	…	14	19,105.5	5.1
X	X	X	…	X	…	…	X	…	12	19,105.6	5.2
X	X	X	…	X	…	X	X	…	13	19,106.0	5.6
X	X	…	X	X	…	X	X	…	15	19,106.1	5.7
X	X	…	X	…	…	…	…	…	11	19,112.9	12.5
X	X	X	…	…	…	…	…	…	10	19,113.7	13.3
X	X	…	…	X	…	…	…	…	10	19,114.4	14.0
X	X	…	…	X	…	…	…	…	10	19,114.4	14.0
X	X	…	…	…	…	…	…	…	9	19,126.5	26.2

*Note*: Sex and species (*Sp*) were included in all models as fixed effects and individual, winter and farm where most fixes were recorded as random intercept terms.

Abbreviations: ΔAIC, difference in Akaike information criterion between models; *Exp*, the cumulative number of days an individual has experienced shooting disturbance on within a winter (continuous); *k*, the number of parameters estimated; *Shot*, categorical variable modeled with either *2 levels* (disturbed and undisturbed) or *3 levels* (undisturbed, disturbed in field, and disturbed nearby); *Sp*, two‐level factor indicating the species, GWfG, or GBG.

### Prediction 4: Differences in time‐activity budgets (GWfG‐only)

To assess the effect of shooting disturbance on time‐activity budgets of GWfG only (as there were no training data for GBG) we used accelerometer data that had been classified, using a random forest model, into four behaviors: walking, grazing, stationary (alert and resting) and flying. The full methodology can be found in Appendix S1: Section S2 but follows previous approaches (Nathan et al., 2012; Weegman et al., 2017). We used a dataset where videoed behaviors were associated with accelerometer bursts from tagged GWfG (walking = 376, grazing = 1689 alert = 472; resting = 287; and flying = 1753); 70% of this data were used to train a random forest model and the remaining 30% were used to test the overall classification accuracy of the model (0.985 [95% CI: 0.977, 0.991]).

We calculated the proportion of bursts classified as grazing, stationary, and flying for each goose‐day and goose‐night. We removed any individuals that had data from less than 10 days in a winter (*n* = 6371 goose day/nights retained). We then modeled variation in the proportion of each behavior in each goose‐day/night using a generalized mixed effects model with a beta binomial error structure (logit‐link function) (Equation 3). Shooting takes place only during the day, goose‐nights were therefore classified as “shooting disturbed” if the individual had experienced a shooting event during the previous day. The global model was fit with a first‐order autoregressive covariance structure (AR1) and included the following fixed predictor effects: (1) shooting disturbance (*Shot*) as a two‐level factor (shooting disturbed and undisturbed); (2) day or night (*Night*) as a two‐level factor (day and night); (3) integer number of days since 1st November as a quadratic term (*Date*); and (4) the interaction between Shooting disturbance (*Shot*) and day or night (*Night*). We also fit individual bird ID (*i*) and winter (*w*) as random intercept terms. The full model took the following form:

$${\text{bursts}}_{\mathrm{iwd}}\sim \text{Binomial}\left(p_{\mathrm{iwd}},n_{\mathrm{iwd}}\right),$$

$$p_{\mathrm{iwd}}\sim \text{Beta}\;\left(a_{\mathrm{iwd}},b_{\mathrm{iwd}}\right),$$

$$a_{\mathrm{iwd}}=\theta \times \pi_{\mathrm{iwd}},b_{\mathrm{iwd}}=\theta \times \left(1-\pi_{\mathrm{iwd}}\right),$$

(3) $$\text{logit}\left(\pi_{\mathrm{iwd}}\right)=\alpha +\beta_{1}{\text{Shot}}_{\mathrm{iwd}}+\beta_{2}{\text{Night}}_{d}+\beta_{3}\left({\text{Shot}}_{\mathrm{iwd}}*{\text{Night}}_{d}\right)+\beta_{4}{\text{Date}}_{\mathrm{iwd}}+\beta_{6}{\left({\text{Date}}_{\mathrm{iwd}}\right)}^{2}+\varepsilon_{\mathrm{iw}},\varepsilon_{\mathrm{iw}}\sim N\left(0,\sigma^{2}\right)$$

where ${\text{bursts}}_{\mathrm{iwd}}$ is the number of bursts classified as a specific behavior for individual *i*, in winter *w*, for the day or night *d*; $n_{\mathrm{iwd}}$ is the total number of bursts; θ is the dispersion parameter (estimated by the model); α is the intercept; the β's are the coefficients of fixed effects; and $\varepsilon_{\mathrm{iw}}$ denotes crossed random effects for each winter *w* and individual *i*. All predictor variables, besides the Shot*Night interaction, were to control for seasonal and between‐winter effects on behavior. Therefore, we compare the global model in Equation (3), with models with the interaction term removed and another where the interaction and shot terms were both removed.

### Prediction 5: Differences in daily energy expenditure

We tested whether shooting disturbance influenced daily energy expenditure, measured using overall dynamic body acceleration (ODBA) (Equation 4). ODBA can be derived from tri‐axial accelerometer data and is a proven proxy for energy expenditure (Qasem et al., 2012). It is calculated for each burst, *j*, across the three axes (*x*, *y*, and *z*) using the formula:

(4) $${\text{ODBA}}_{j}=\frac{\sum_{i=1}^{n}\left|x_{i}-\bar{x}\right|+\left|y_{i}-\bar{y}\right|+\left|z_{i}-\bar{z}\right|\;}{n}$$

where *x*_*i*_ represents the *i*‐th component and *x¯* the mean of all *n* samples within burst *j* for the *x* axis, and likewise for *y* and *z*.

We calculated the total ODBA for each individual during the day by summing all ODBA values. We removed individuals with less than 10 different days of data throughout an entire winter (*n* = 3985 goose days retained). We modeled variation in total ODBA using a generalized mixed effects model with a gamma error structure (log‐link function) (Equation 5). We fitted a first‐order autoregressive covariance structure (AR1), and because the number of accelerometer bursts varied between days, we fitted the number of bursts as a fixed effect. The global model included the following fixed effects: (1) Shooting disturbance (*Shot*) as a two‐level factor (shooting disturbed and undisturbed); (2) Species (*Sp*); (3) the two‐way interaction between Shooting disturbance (*Shot*) and Species (*Sp*); (4) Sex (*Sex*); (5) integer number of days since 1st November as a quadratic term (*Date*); and (6) the number of accelerometer bursts recorded that day (*N*). We also fitted individual bird ID (*i*) and winter (*w*) as random intercept terms. The full model took the following form:

$${\text{ODBA}}_{\mathrm{iwg}}\sim \text{Gamma}(\mu_{\mathrm{iwg}},\gamma_{\mathrm{iwg}}),$$

(5) $$\log(\mu_{\mathrm{iwg}})=\alpha +\beta_{1}{\text{Shot}}_{\mathrm{iwg}}+\beta_{2}{\mathrm{Sp}}_{g}+\beta_{3}({\text{Shot}}_{\mathrm{iwg}}*{\mathrm{Sp}}_{g})+\beta_{4}{\mathrm{Sex}}_{\mathrm{iwg}}+\beta_{5}{\text{Date}}_{\mathrm{iwg}}+\beta_{6}{\left({\text{Date}}_{\mathrm{iwg}}\right)}^{2}+\beta_{7}N_{\mathrm{iwg}}+\varepsilon_{\mathrm{iw}},\varepsilon_{\mathrm{iw}}\sim N\left(0,\sigma^{2}\right)$$

where ${\text{ODBA}}_{\mathrm{iwg}}$ is the total daily ODBA for individual *i*, in winter *w*, for goose species, *g*; α is the intercept; the β's are the coefficients of fixed effects; and $\varepsilon_{\mathrm{iw}}$ denotes crossed random effects for each winter *w* and individual *i*. We expect ODBA to have a nonlinear relationship with date due to foraging rates peaking in late winter when resources are most depleted, therefore it was fit as a quadratic term. All predictor variables, besides the Shot*Sp interaction, were to control for seasonal and between winter effects on ODBA. Therefore, we compare the global model in Equation (5), with four a‐priori other models, first removing the Shot*Sp interaction, then *Shot* and *Sp* in turn and finally removing both.

### Prediction 6: Differences in habitat selection

To determine whether daily habitat use is influenced by shooting disturbance we assessed if habitat selection of individual geese varied according to shooting exposure. This was firstly assessed between days, comparing habitat selection on days when individuals were disturbed by shooting with undisturbed days. Secondly, we assessed habitat selection within disturbed days, comparing selection before and after shooting disturbance. To reduce the impact of roosting site selection we limited our analysis to daytime GPS fixes. All GPS fixes were classified as “shooting” or “nonshooting” depending on whether the individual was disturbed by shooting on a given day (see *Prediction 2: Classification of shooting disturbance*). Then, within shooting disturbed days, fixes were classified as “pre‐shooting” or “post‐shooting” according to whether the timestamp of the fix occurs before or after the timestamp of the shooting event. Additionally, tracking data was restricted to 2015–2020 as this was the period for which Islay‐specific habitat data were available, and we removed all individual with <5 locations within a given day for each disturbance classification.

We used resource selection models to compare habitats used by geese to those available in the wider landscape (Aarts et al., 2012). For all analysis we created a set of random locations that individuals could have feasibly used (pseudoabsences) at a ratio of 10 random locations for every used GPS fix. Pseudoabsences were generated within the 100% minimum convex polygon for each species across all individuals using the “random_points” function in the R package *amt* (Signer et al., 2019). This allowed us to capture the full composition of available habitats within the foraging range of each species. To assess differences in habitat selection between disturbed and undisturbed days all fixes (used and pseudoabsences) were then classified as one of seven habitat categories (improved grassland, other grassland, arable, saltmarsh and coastal, bog, freshwater and other) using the 20 m × 20 m resolution landcover map of Britain from the same year (see Appendix S2: Figure S1) (Morton et al., 2020). For models comparing pre‐ and post‐shooting habitat selection within days, habitats with less than 50 real GPS fixes in both pre‐ and post‐shooting classes were grouped and classified as “other.” The following habitat classes remained for GBG: arable, improved grassland, saltmarsh and coastal, and other. While for GWfG, arable, improved grassland, other grassland, and other remained.

For both species, resource selection models were performed using generalized linear models with a binomial error structure (logit link function). Used GPS fixes were assigned a weighting of 10 and pseudoabsences a weighting of 1 (Muff et al., 2020). We modeled habitat use (binomial response variable, used = 1, pseudoabsence = 0) at two temporal scales and separately for each species. For comparisons of habitat use between shooting disturbed and nonshooting days, habitat use was modeled in response to two explanatory variables: habitat type (seven‐level factor) and shooting disturbance (two‐level factor), as well as their two‐way interaction. Winter was not included as a variable as habitat availability has been largely consistent for the duration of the study. Differences in habitat use before and after shooting disturbance within a day were assessed by modeling habitat use in response to three explanatory variables: habitat type (four‐level factor), shooting period (two‐level factor, pre‐ and post‐ shooting), and distance to road (scaled continuous variable) along with the three‐way interaction. Distance to the nearest road was included as a predictor as shooting is often conducted close to roads (see Appendix S2: Figure S2 for road network map). In all models, a significant interaction term between habitat type and shooting disturbance suggests habitat selection varies with shooting disturbance. Random intercepts to account for repeated individual measures were not fit as this represents the constant ratio of used locations to pseudoabsences (Raymond et al., 2015).

For all habitat selection models, model fit was assessed by creating a receiver operating characteristic curve (ROC) from which we calculated the area under the curve (AUC). AUC greater than 0.5 indicates a better than random model fit, with optimal support for values >0.8 (perfect model fit; Fielding & Bell, 1997; Zweig & Campbell, 1993). Additionally, predictive power, sensitivity, specificity, and accuracy were derived from a confusion matrix based on the original data (Appendix S2: Tables S1–S4) (Warwick‐Evans et al., 2016).

## RESULTS

### Prediction 1: Displacement due to shooting

There was a negative relationship between the change in step length (between time period, *t*, and control time period, *t‐1*) and the distance from the shooting event for GBG β = −224.3 [95% CI: −334.2, −116.9] and GWfG β = −90.0 [95% CI: −138.6, −41.7] (all CIs are 95% hereafter) (Figure 3). This suggests that the increase in movement due to shooting decays as birds became further from the shooting event. The intercept estimate gives the change in step‐length in meters when an individual was 0 m from the shooting event and likely equates to birds being shot at or in the same field as the target flock. For GBG the intercept was β = 1711.0 [CI: 893.6, 2553.2] and for GWfG it was β = 630.6 [CI: 227.9, 986.7]. To determine at what distance from the shooting event there appeared to be no significant change in step‐length, we extracted the point at which the lower 95% CI for the relationship between change in step‐length and distance to shooting event first crossed the *y* = 0 line; for GBG this value was 1184 m and for GWfG it was 664 m. Overall, these results suggest that compared with GWfG, GBG have a larger increase in movement when directly exposed to shooting, and this heightened response persists at distances further from the shooting source.

### Prediction 2: Classification of shooting disturbance

To understand how often individuals are disturbed by shooting within a winter, we quantified the average number of times per day that individuals were within 1184 m (GBG) or 644 m (GWfG) of the shooting field centroid immediately prior to when shooting took place (Appendix S2: Figure S3). For both species the maximum rate of shooting disturbance was low (<0.7 events per goose‐day). GWfG showed more variability in disturbance rate likely due to a greater number of sampled winters which differ in annual shooting intensity, with higher historic shooting intensity in years prior to tracking of GBG. The mean number of disturbance events per day was 0.20 (SE = 0.045) for GWfG, and 0.09 (SE = 0.015) for GBG.

### Prediction 3: Differences in daily foraging distance

**FIGURE 4:**
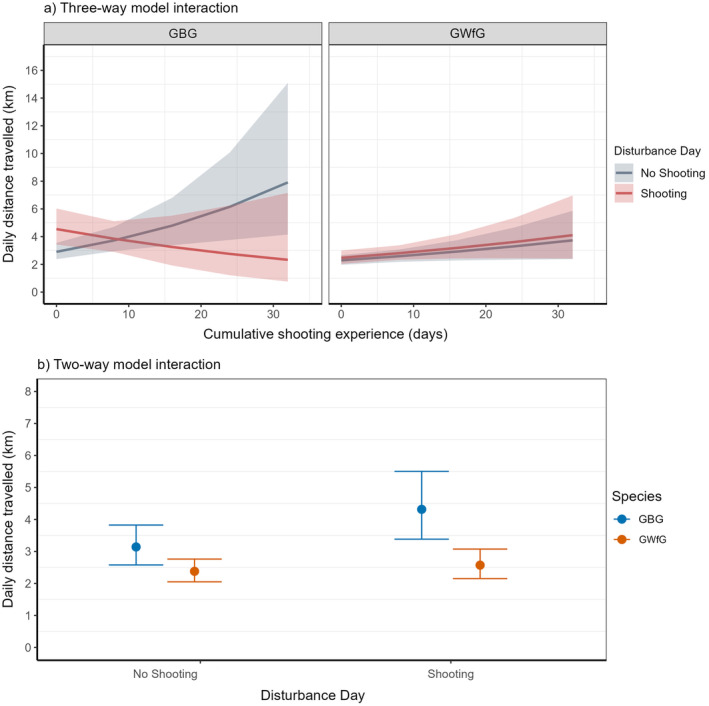
The total distances traveled on days individual geese were not exposed to shooting (no shooting) compared with days on which they were exposed to one or more shooting events (shooting). (a) Representing the three‐way model interaction between shooting disturbance, species, and cumulative shooting experience (number of unique days an individual is exposed to shooting disturbance within a winter). (b) The two‐way interaction between species and shooting disturbance. Back transformed model estimates and 95% CIs are depicted from the best Akaike information criterion supported model (Table 1). Total distance traveled is calculated by summing the point‐to‐point distances between GPS fixes within a day for a single individual. GBG, Greenland barnacle geese; GWfG, Greenland white‐fronted goose.

The top seven models were within 3.9 corrected AIC (AIC_c_) of the top model and all included significant effects of disturbance, species, and their interaction (with disturbance modeled as either a two‐level or a three‐level factor). Here we report the results from the highest ranked model which included the three‐way interaction between species, individual shooting experience and shooting disturbance as a two‐level factor β = 0.22 [CI: 0.06, 0.37] (Table 1, conditional *R* ^2^ = 0.57, marginal *R* ^2^ = 0.05). For GBG, travel distances were consistent regardless of prior experience on shooting days, but on nonshooting days individuals with more prior experience of shooting had greater daily travel distances, while GWfG showed no significant difference in daily travel distances regardless of prior experience or shooting disturbance (Figure 4a). For GBG, daily foraging distance increased significantly on days when individuals experienced shooting disturbance from 3.14 km [CI: 2.58, 3.83] on undisturbed days to 4.32 km [CI: 3.38, 5.50] (Figure 4b). GWfG showed a small insignificant increase from 2.38 km [CI: 2.05, 2.76] on undisturbed days to 2.57 km [CI: 2.15, 3.07] on shooting‐disturbed days. There were no apparent differences between sexes.

### Prediction 4: Differences in time‐activity budgets (GWfG‐only)

When testing for the effect of shooting on the time spent stationary, *bursts*_*stationary*_, the top AIC ranked model did not contain the Shot*Night interaction term or *Shot* term (Table 2). The second ranked model (difference in AIC from top model [ΔAIC] = 1.05) included the *Shot* term but not the Shot*Night interaction term. In this second ranked model the parmeter estimate for the *Shot* term overlapped zero, β = 0.036 [CI: −0.036, 0.109]. This result indicates that there was little support for shooting disturbance influencing the time spent stationary. When testing for the effect of shooting on the time spent grazing, *bursts*_*grazing*_, the top ranked model contained the *Shot* term but not the Shot*Night interaction term (Table 2). This suggests that shooting disturbance may influence grazing but the effect did not differ between day and night. The parameter estimate for the *Shot* term (undisturbed as the reference level) was β = −0.073 [CI: −0.147, 0.001], indicating a 7.3% decrease in grazing time, from 44.6% to 42.8% of all bursts, when disturbed by shooting. However, the CI of this estimate marginally spanned zero so there is a low degree of certainty in this interpretation. When testing for the effect of shooting on time spent flying, *bursts*_*flying*_, the *Shot* term and the Shot*Night interaction term were in the top ranked model. Although the AIC for a model with neither term was only marginally higher  (ΔAIC = 0.36). In the top ranked model the parameter estimate for the *Shot* term (undisturbed night as reference level) was β = 0.089 [CI: 0.004, 0.175] and for the Shot*Night interaction term was β = *−*0.110 [CI: −0.245, 0.025]. This indicates no change in flight duration during the day but an 8.9% increase in flight during the night when disturbed by shooting in the previous daytime, increasing from 5.3% to 5.8% of bursts.

**TABLE 2:** Model selection table to understand the effect of shooting disturbance (*Shot*) on the proportion of accelerometer bursts classified as three different behaviors: grazing, stationary and flying.

Response variable	*Date*	*Date* ^*2*^	*Night*	*Shot*	Shot*Night	*k*	AIC	ΔAIC
*Bursts*_*grazing*_	1.458 [−1.017, 3.933]	−9.893 [−12.08, −7.696]	−2.170 [−2.140, −2.200]	−0.073 [−0.147, 0.001]	…	12	48,616.18	0
*Bursts*_*grazing*_	1.461 [−1.014, 3.936]	−9.890 [−12.09, −7.693]	−2.167 [−2.138, −2.197]	−0.049 [−0.143, 0.046]	−0.057 [−0.197, 0.082]	11	48,617.53	1.35
*Bursts*_*grazing*_	1.476 [−0.998, 3.940]	−9.879 [−12.08, −7.682]	−2.170 [−2.140, −2.199]	…	…	10	48,617.88	1.70
*Bursts*_*stationary*_	5.545 [2.819, 8.088]	7.886 [5.567, 10.21]	2.365 [2.336, 2.394]	…	…	10	47,992.06	0
*Bursts*_*stationary*_	5.464 [2.830, 8.099]	7.892 [5.572, 10.21]	2.365 [2.336, 2.394]	0.036 [−0.038, 0.109]	…	11	47,993.15	1.09
*Bursts*_*stationary*_	5.466 [2.831, 8.101]	7.894 [5.574, 10.21]	2.366 [2.336, 2.396]	0.047 [−0.058, 0.152]	−0.020 [−0.152, 0.113]	12	47,995.06	3.00
*Bursts*_*flying*_	−2.707 [−6.056, 0.643]	1.838 [−0.689, 4.374]	−0.633 [−0.662, −0.604]	0.089 [0.004, 0.175]	−0.110 [−0.245, 0.025]	12	27,986.46	0
*Bursts*_*flying*_	−2.748 [−6.112, 0.617]	1.8303 [−0.737, 4.342]	−0.638 [−0.667, −0.610]	…	…	11	27,986.82	0.36
*Bursts*_*flying*_	−2.709 [−6.059, 0.640]	1.829 [−0.707, 4.365]	−0.638 [−0.667, −0.610]	0.048 [−0.022, 0.117]	…	10	27,987.04	0.58

*Note*: The days since November 1 as a quadratic term (*Date* ^*2*^) and a day/night categorical variable were included in all models as fixed effect and individual ID as a random intercept term. For each variable the parameter estimate and 95% CIs are given if it is included in a model. Models were ranked using Akaike information criterion (AIC).

Abbreviations: *k*, the number of parameters estimated in the model; *Night*, fixed effect variable indicating if data is from the day or night (two‐level factor); *Shot*, fixed effect variable indicating if birds were exposed to shooting on a given day (two‐level factor).

### Prediction 5: Differences in daily energy expenditure

The top model (marginal *R* ^2^ = 0.864) did not contain the terms of interest, species (*Sp*) or shooting exposure (*Shot*), suggesting that there was no effect of shooting disturbance on total daily ODBA, and that it did not differ between species (Appendix S2: Table S5). There was a significant quadratic effect of time of year across all models, β = −3.119 [CI: −5.193, −1.026] in the top ranked model, where ODBA peaked later in the winter before decreasing slightly in March. This peak coincides with increasing day lengths and fuel loading for migration when feeding is expected to increase. There was no consistent sex effect with estimates always overlapping zero, β = −0.015 [CI: −0.154, 0.125] in the top ranked model.

### Prediction 6: Differences in habitat selection

**FIGURE 5:**
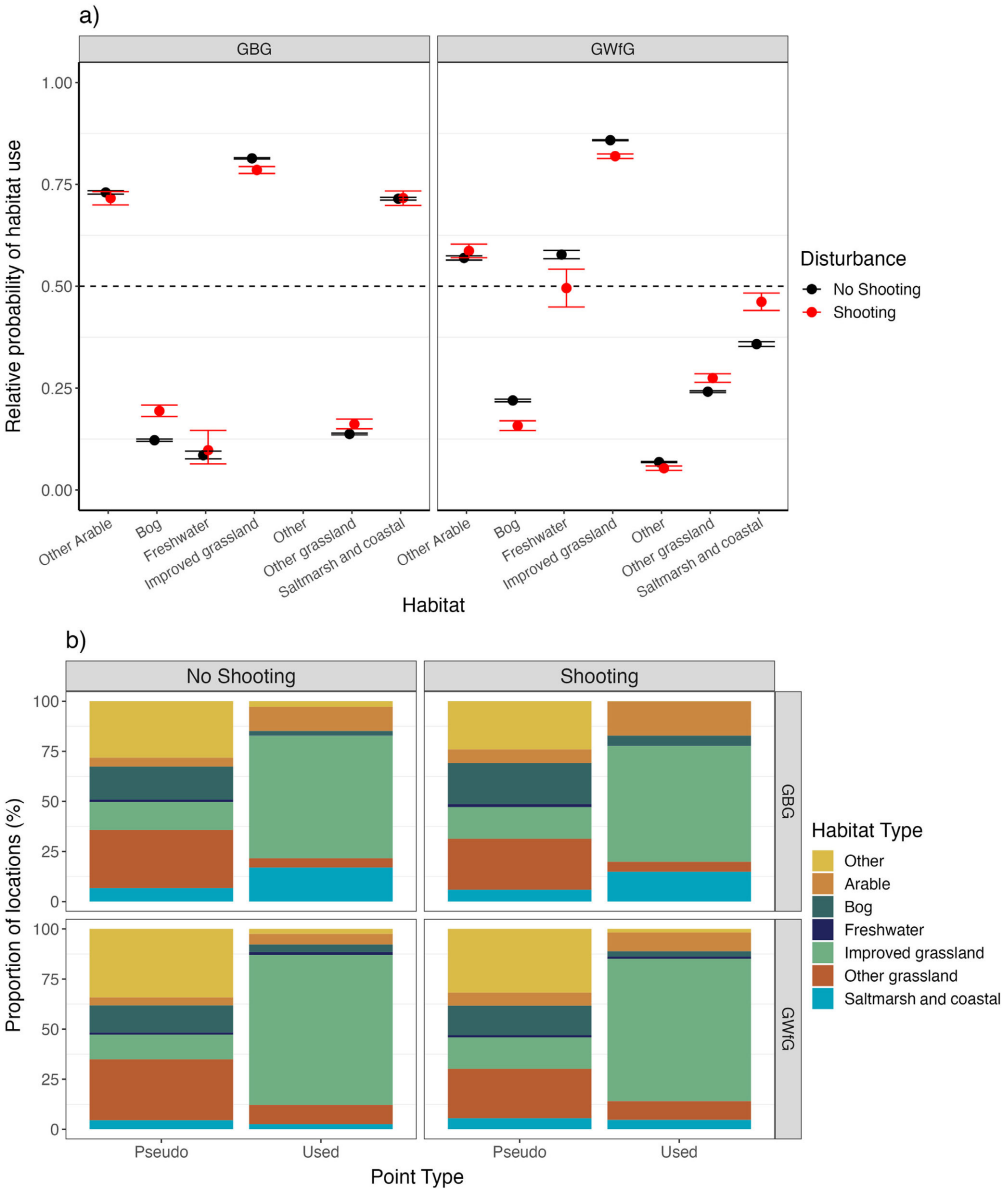
(a) Estimates and 95% CIs from the best performing resource selection model ranked by Akaike information criterion (Appendix S2: Table S1), to understand habitat selection for Greenland barnacle geese (GBG) and Greenland white‐fronted goose (GWfG) on days individuals were exposed to shooting or not (shooting or no shooting). Models estimate the relative probability of a given foraging point being a real feeding location rather than a pseudo‐absence in response to one of seven habitat classes as a result of one of two disturbance classifications (no shooting disturbance and shooting disturbance). A probability of 0.5 indicates that birds use a habitat in proportion to its availability. Values >0.50 indicate selection for that habitat. (b) Proportion of goose foraging locations (used) versus randomized pseudo‐absences (pseudo) according to the seven main habitat classes.

Habitat selection between days was modeled separately for each species, and in both instances the most parsimonious model included the two‐way interaction between shooting disturbance and habitat (Appendix S2: Table S1). For both species, habitat selection differed between shooting and nonshooting disturbed days. For GBG, patterns in overall habitat selection between days were consistent regardless of shooting, such that improved grassland β = 3.21 [CI: 2.64, 3.90], arable β = 3.32 [CI: 2.75, 4.01], and saltmarsh β = 3.40 [CI: 2.82, 4.09] habitats were strongly selected for in relation to their availability (probability of use >0.5). However, the selection for these habitats was lower on shooting days, while selection for bog β = 3.94 [CI: 3.36, 4.63] and other grasslands β = 3.58 [CI: 3.01, 4.28] appear to increase (Figure 5; Appendix S2: Table S2). By contrast, while GWfG showed similar selection for improved grassland β = −0.02 [CI: −0.17, 0.09] and arable habitats β = 0.33 [CI: 0.21, 0.47] regardless of shooting disturbance, saltmarsh/coastal habitats β = 0.70 [CI: 0.56, 0.84] and freshwater habitats β = −0.07 [CI: −0.28, 0.15] showed notable changes in selection between shooting disturbed and nonshooting days (Figure 5; Appendix S2: Table S2).

**FIGURE 6:**
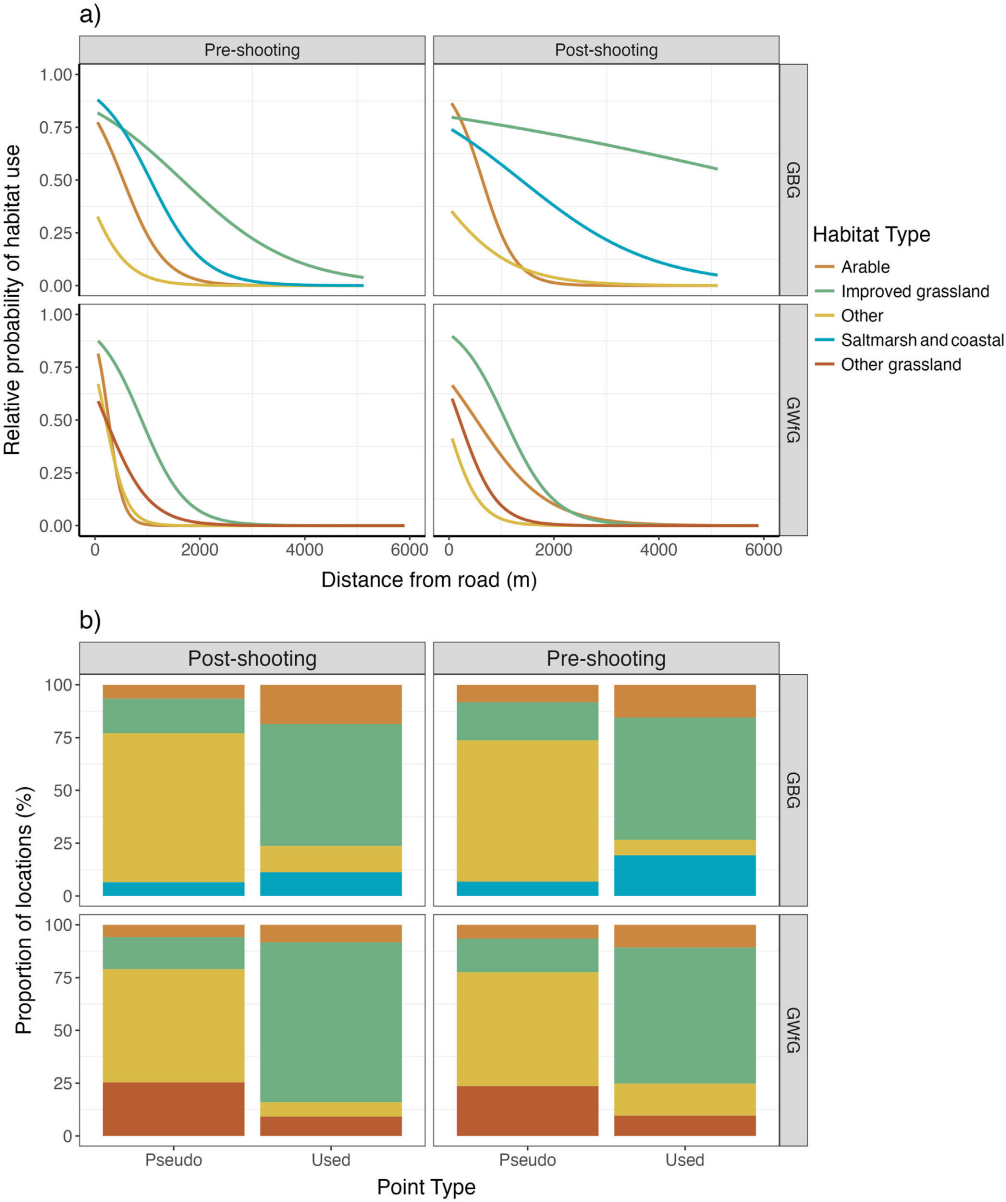
(a) The relative probability of habitat use, compared with availability, in Greenland barnacle geese (GBG) and Greenland white‐fronted goose (GWfG). The relative intensity of use of different habitats within shooting disturbed days is compared before and after exposure with a shooting event and as a function of distance to the nearest public road (in meters). Curves represent the mean estimates from the best performing model ranked by Akaike information criterion for GWfG and GBG (Appendix S2: Tables S2 and S3). (b) Proportion of goose foraging locations (used) versus randomized pseudo‐absences (Pseudo) according to four main habitat classes grouped with a minimum of 50 “used” fixes per habitat and shooting class.

The most parsimonious model for comparing habitat selection pre‐ and post‐shooting within shooting disturbed days was also the full model and included the three‐way interaction between habitat classification, shooting disturbance and distance to road (Appendix S2: Tables S3 and S4). This means that habitat selection was influenced by distance to the nearest road, varied according to the habitat type and differed between pre‐ and post‐shooting time periods. For both species across all habitats, relative probability of habitat use declined with increasing distance from roads. However, in GBG the relative probability of use of improved grassland remained high at greater distances from roads following disturbance than prior to shooting β = 0.42 [CI: 0.06, 0.78]. This suggests that shooting disturbance changes habitat selection of GBG in relation to roads but not for GWfG (Figure 6).

## DISCUSSION

Combining biologging data with shooting records at fine spatiotemporal resolutions has enabled a comparison of the movement and behavioral responses to shooting for a target and nontarget species in sympatry. While we detected behavioral responses in GBG and GWfG, the effect of shooting disturbance was more pronounced in GBG, which are targeted as part of shooting management. Our study highlights the importance of accounting for behavioral responses within shooting management strategies and we identify several recommendations for management strategies aimed at mitigating human−wildlife conflict.

### Interspecific differences in disturbance response

The distances from shooting at which a movement response is detectable was higher for GBG (1184 m) than for GWfG (644 m). This greater flight initiation distance in the target species has been shown previously (Laursen et al., 2005; Madsen et al., 1998) and is greater than for human presence alone, which has been estimated at 47.4 m for *Anseriformes* geese (Livezey et al., 2016) and at 211 m for Brent geese *Branta bernicla* in open landscapes (Madsen, 1988). We also found increased daily travel distances and shifts in habitat selection that were present in GBG but not observed in GWfG on shooting disturbed days. We suggest these interspecific differences are due to any combination of: (1) higher perceived risk of mortality associated with shooting in GBG; (2) larger flock sizes in GBG and; (3) exposure to stronger shooting stimuli in GBG.

The strength of a behavioral response can be influenced by the level of mortality risk associated with a stimulus. The risk‐disturbance hypothesis predicts stronger behavioral responses when the perceived mortality risk is greater (Frid & Dill, 2002). Shooting can cause stronger behavioral responses in the target species as the nontarget species never experiences significant negative reinforcement, in the form of observed mortality, when exposed to shooting disturbance (Bejder et al., 2009; Livezey et al., 2016; Thiériot et al., 2012). Only GBG are targeted as part of the ISGMS and therefore could establish an association between shooting disturbance and increased mortality risk which should not occur in GWfG. This could explain the lower immediate movement response to shooting in GWfG. We also found that shooting disturbance only significantly increased total daily movement in GBG (Figure 4b). This could be due to a larger initial escape flight in GBG and additional escape flights post‐shooting due to increased sensitivity to other disturbance stimuli (Dooley et al., 2010; Laursen et al., 2005). For instance, after periods of shooting disturbance, Brent geese initiated flight at larger distances compared with nonshooting periods (Madsen, 1988). As GWfG may not experience the same degree of fear conditioning and have little association between shooting disturbance and mortality risk, then shooting may elicit a dampened behavioral response.

Secondly, interspecific differences in flock size may influence the likelihood that any individual takes flight due to shooting disturbance. Group size is positively correlated with the flight initiation distance from anthropogenic disturbance in a range of taxa, for example, Common pochard *Aythya ferina* (Batten, 1977; Mori et al., 2001), migratory shorebirds (Burger & Gochfeld, 1991), and Vervet monkeys *Chlorocebus pygerythrus* (Mikula et al., 2018). This is generally because larger groups have a higher likelihood of any one individual taking flight and flushing the entire group, simply due to there being more individuals (Hilton et al., 1999). Consequently, the larger flock sizes of GBG (540 individuals) relative to GWfG (90 individuals) (McIntosh et al., 2023) could lead to GBG exhibiting increased movement at a greater distance from shooting (Burger & Gochfeld, 1991).

Perceived predation risk is associated with apparent predation intensity and lethality (Frid & Dill, 2002; Laundré et al., 2001). Increased perceived predation risk is often associated with closer proximity of an individual to a predator (McCormick & Manassa, 2008), or in our instance, the proximity of a goose to a shooting event (Thurfjell et al., 2013). We found that both species were displaced by shooting when close to a shooting event and that GBG were displaced further, especially at distances closer to shooting. This could arise due to an underestimation of how far GWfG are from a shooting event due to the use of field centroids as the approximate location of a shooting event. According to the ISGMS, GWfG should never be the target of shooting, but they can be in a separate flock in the same field as the shooting event. This could create a bias, such that GWfG were further from shooting events than we estimated.

### Energetic consequences of responding to shooting disturbance

Changes in behaviors after and during disturbance, such as increased flight, reduced foraging time, and increased vigilance, are energetically costly. Without adequate energetic compensation over time this can lead to starvation and/or have downstream effects on migratory performance, reproductive success, and survival (Bauer et al., 2018; Béchet et al., 2004).

For GWfG there are no significant increases in daily travel distances or energy expenditure on days when individuals were disturbed by shooting. There was a small decrease in the proportion of time spent grazing from 44.6% to 42.8% (marginally insignificant) and an increase in the time spent flying during the night from 5.3% to 5.8% (significant). The small decrease in foraging time (if this is indeed a true effect) may occur due to increases in flight and vigilance immediately following disturbance that were too small to detect with our current analysis. The increase in nocturnal flight may be due to individuals having to fly further back to the roost site after being flushed by shooting during the day. This return to roost flight often occurs after sunset as birds remain foraging on agricultural fields past sunset during short winter days. Therefore, any accelerometer bursts from longer roost flights are assigned to night instead of day. As these changes in behavioral habits are small and there is no increase in energy expenditure measured via ODBA, it seems unlikely that GWfG experience an energetic deficit that could not be compensated for.

For GBG, daily travel distances increased when disturbed by shooting but we found no evidence of concurrent increases in ODBA. This could be due to increases in movement not being sufficient to significantly increase ODBA, but this seems unlikely given such a large increase in travel distance. Changes in behavioral allocation (not assessed here in GBG) could explain the lack of change in ODBA if increased vigilance offset ODBA increases due to additional flight. If this were the case then individuals may have to compensate for lost foraging time by increasing foraging duration and rates (Madsen, 2001, Nolet et al., 2016), which could worsen agricultural damage. Given the large increase in daily travel distances in GBG, determining whether compensatory feeding occurs should be a priority of future research to understand both the energetic consequences for GBG and the effectiveness of the goose management scheme to reduce grazing pressure.

On Islay, shooting is restricted to specific fields within large areas of improved grassland providing areas of undisturbed foraging opportunities for both species (Mckenzie & Shaw, 2017). The individuals most frequently exposed to shooting were disturbed once every two days for GWfG or once every four days for GBG. On average though, GWfG were disturbed less than every three days, and GBG less than every 10 days (Appendix S2: Figure S3). An energetic model based on White‐fronted geese *Anser albifrons* found individuals disturbed an additional six times per day (excluding roost flights) would no longer be able to compensate for increased energetic requirements (Nolet et al., 2016) and further exposure would incur fitness costs (Kleist et al., 2018). Previous studies on Islay found that GWfG are disturbed every 6.5 h, equating to 1–2 disturbance events per day (including walkers, cars, farm activity, predators) (Griffin et al., 2020). Therefore, even the GWfG disturbed most frequently by shooting should be able to compensate energetically. It is unknown how often GBG on Islay are disturbed by other stimuli, for example, vehicles and predators, but it is perhaps higher than GWfG, given greater daily travel distances on days not disturbed by shooting (Figure 5). On shooting‐disturbed days this could lead to individuals being in an energy deficit, though these instances are likely to be relatively infrequent.

The energetic model for White‐fronted geese (Nolet et al., 2016) does not account for changes in basal metabolic rate caused by disturbance (Viblanc et al., 2015), meaning that fewer disturbances could lead to energy deficits. In the highest intensity shooting areas on Islay, increases in metabolic rate could reduce fitness (West et al., 2002). However, given the low frequency of disturbance observed in this study, severe fitness effects are unlikely. In addition, energy requirements peak during the pre‐migratory fattening period and birds may therefore be particularly sensitive to shooting disturbance at this time (Handby et al., 2022). For example, shooting disturbance prior to migration has been associated with reduced lipid and protein storage (Pearse et al., 2012), delayed departure dates (Gunnarsson et al., 2006), and poorer departure condition (Inger et al., 2010). Further work could assess the cumulative effect of stress from repeated disturbance on basal metabolic rate, especially because delayed migratory departure of disturbed individuals (Bauer et al., 2018; Béchet et al., 2004) could extend goose−agricultural conflict.

### Distributional consequences of responding to shooting disturbance

Both species displayed a consistent preference for improved grassland sites regardless of shooting disturbance (Figure 5). However, GBG exhibited a greater relative probability of use for fields further from roads after experiencing shooting disturbance, compared with before disturbance (Figure 6). This is likely due to shooting taking place on fields closer to roads and GBG redistributing to improved grassland sites with lower background disturbance that tend to be further from roads, a behavior observed in other taxa (Béchet et al., 2003; Grignolio et al., 2011; Jensen et al., 2016; Montràs‐Janer et al., 2020; Thorsen et al., 2022). This redistribution risks displacing agricultural damage to other agricultural land which then requires further management (Tombre et al., 2013). Prolonged disturbance could also lead to larger scale redistributions, such as individuals emigrating to the nearby islands of Tiree, Coll, and Uist (where GBG populations have recently increased, although this has not been linked to Islay management) (Mitchell & Hall, 2020). Monitoring of site exchange between and within years would be required to determine if such redistributions were occurring.

GWfG do not appear to show any change in habitat selection within days of experiencing shooting disturbance (Figure 6), which may be due to the lower sensitivity of GWfG to shooting disturbance (discussed above) (Laursen et al., 2005). However, as GWfG are not targeted, their disturbance is contingent on proximity to GBG when foraging. Their difference in habitat selection between days when they experience shooting disturbance and not, may just reflect differences in habitats surrounding designated shooting fields. For example, GWfG have a higher relative probability of using saltmarsh on nonshooting days (Figure 5) which may be due to some shooting designated fields being close to area of saltmarsh.

## MANAGEMENT IMPLICATIONS AND RECOMMENDATIONS

GBG seek refuge following shooting disturbance as birds show strong selection for improved grassland further from roads where disturbance is typically lowest. From our data, it is unlikely that this results in negative fitness effects, but there may be compensatory feeding as movement is increased on average by 38% on days an individual is disturbed by shooting. If compensatory feeding was to occur, this could increase per capita food consumption as individuals recoup energetic losses from disturbance responses. If frequent enough, this could dampen efforts to limit damage to agricultural grassland (the overall goal of the ISGMS). The current management scheme can shoot over up to 40% of agricultural land within the scheme in any year (not including agricultural land outside of the scheme and nature reserves), providing at least 60% as unstructured refuge areas (Mckenzie, 2014). Disturbance effects can extend far beyond where the shot is fired and could limit the effectiveness of current unstructured refuges. For example, shooting occurs adjacent to an important refuge area, the Gruinart Flats Special Protected Area. To ensure refuge areas are effective, one approach suggests their diameter should be three times the size of the escape flight initiation distance (Fox & Madsen, 1997). Using this method, our results indicate refuges on Islay would have a minimum diameter of 3.54 km for GBG and 1.9 km for GWfG and encompass areas of improved grassland and coastal saltmarsh for foraging and roosting. Multiple refuge areas connected as a useable network can help to boost their effectiveness (Chudzińska et al., 2016; Madsen, 1998; Madsen et al., 1998; Summers & Hillman, 1990). Overall, a well‐structured and connected refuge area design could prevent further disturbance to birds following shooting, minimize compensatory feeding and direct grazing away from reseeded grassland that the goose scheme aims to protect and shoots over. However, the current distribution of shooting and refuge fields within the scheme has come about largely due to practicalities of topography and farm management, and shooting disturbance events were found to be relatively infrequent.

Shooting management should mitigate human−wildlife conflict while maintaining viable wild populations and limiting the indirect effects of disturbance on all species. This necessitates the incorporation of spatiotemporal displacement and fitness effects into the development of shooting strategies, and subsequent monitoring. Our study provides an example of the effectiveness of using biologging to assess the indirect impact of shooting management and highlights the importance of incorporating assessments of unseen behavioral responses and nontarget species. In light of our results, we make three management recommendations:

1. Assessing the behavioral and movement consequences of disturbance should ideally be monitored before, during, and after shooting, over short and long temporal scales. This ensures that the immediate and long‐term consequences of shooting can be assessed and that behavioral changes after disturbance do not exacerbate conflict or overly harm target populations. Biologging provides the ideal tool to carry out this longitudinal monitoring of individuals irrespective of where they move to.
2. Assessment of the effects of disturbance on nontarget species and their responses should be done independently as responses can be species‐specific, as shown here. The responses of vulnerable nontarget species to shooting disturbance, combined with their distribution, may guide where shooting is carried out in a landscape and where refuge areas should be located to function effectively for nontarget species.
3. Local and regional monitoring of populations should extend beyond the immediate vicinity of active shooting to identify over what scales spatial and temporal displacement is occurring. From a management perspective, this can identify scenarios where conflict is being displaced or identify where refuges could be created to hold displaced individuals and minimize further conflict. From a conservation perspective it enables the fitness of displaced individuals to be monitored to assess long‐term population viability.

## AUTHOR CONTRIBUTIONS

Aimée L. S. McIntosh, Luke Ozsanlav‐Harris, Geoff M. Hilton, Larry R. Griffin, Jessica M. Shaw, and Stuart Bearhop conceived the study and analysis. Luke Ozsanlav‐Harris and Aimée L. S. McIntosh contributed equally to the analysis and initial draft. Larry R. Griffin and Stuart Bearhop lead biologging device deployment assisted by Luke Ozsanlav‐Harris, and Aimée L. S. McIntosh. Jessica M. Shaw provided data from NatureScot. All authors reviewed and approved the final manuscript.

## CONFLICT OF INTEREST STATEMENT

The authors declare no conflicts of interest.

## Supporting information


Appendix S1.

Appendix S2.

## Data Availability

Data and code (Ozsanlav‐Harris et al., 2024) are available in Zenodo at https://zenodo.org/doi/10.5281/zenodo.13383185 where we provide all derived data required to run our analysis (e.g., timestamps, tag identifier codes, step lengths and habitats associated with each GPS fix) and locations rounded to one decimal place. A dynamic set of data and code can be found on GitHub at https://github.com/LukeOzsanlav/Islay_ShootingDisturbance. Raw GPS data supporting this research are sensitive and not available publicly but are available to qualified researchers; use of these data will be restricted to research, and users will not be allowed to distribute the data. Raw GPS data for Greenland white‐fronted geese are owned by the Wildfowl and Wetland Trust and are available by contacting the Wildfowl and Wetlands Trust (enquiries@wwt.org.uk) and requesting the GPS data from Greenland white‐fronted geese tagged on Islay up to the year 2021. Raw GPS data for Barnacle geese are available by contacting Aimée L. S. McIntosh (a.l.s.mcintosh@exeter.ac.uk).
