# Supplementary material for: Contrasting effects of shooting disturbance on the movement and behavior of sympatric wildfowl species
Source: Ecol Appl. 2024 Oct 25;34(8):e3032. doi: 10.1002/eap.3032 (PMC11610659; doi:10.1002/eap.3032)
Supplement: Supplementary file 1 — Appendix S1. [file EAP-34-e3032-s002.pdf]

# Appendix S1

## Contrasting effects of shooting disturbance on the movement and behavior of sympatric wildfowl species

Luke Ozsanlav-Harris, Aimée L.S. McIntosh, Larry R. Griffin, Geoff M. Hilton, Lei Cao, Jessica M. Shaw & Stuart Bearhop

**Journal**: Ecological Applications

## Section S1: GPS tracking

Geese were caught using cannon nets at baited sites across Islay. All birds were sexed post-capture via cloacal examination. GBG were captured between October and February in winters 2019/2020 and 2020/2021. Three tag types were deployed: nanoFix GEO+RF (Pathrack Ltd, Otley, UK, 15g, n=13), Ecotone GPS-GSM (Ecotone, Gydnia, Poland, 25g, n=9) and Ornitela OT-NL40 GPS-GSM (Ornitela, Vilnius, Lithuania, 19g, n=16) tags. 38 geese were fitted with tags over both winters (n=29 winter 2019/2020, n=9 winter 2020/2021). Tags were deployed on birds of both sexes with a large head and bill sizes (> 84.5mm) to ensure the suitable tag fit. However, this size constraint meant most tags were fitted on males. Given that GBG form long-term pair bonds with close associations, our results are unlikely to be affected by sex-bias. GWfG were captured between October and February every winter from 2013/2014 until 2020/2021. Similarly, three transmitter types were deployed: Ornitela model N38 GPS-GSM (Ornitela, Vilnius, Lithuania, c37g, n = 24), Ecotone GPS-GSM (Ecotone, Gydnia, Poland, c24g, n = 41) and Ecotone GPS-UHF (Ecotone, Gydnia, Poland, c24g, n = 29). Tags were deployed on both sexes (75 females, 19 males), again results are unlikely to be affected by sex-bias due to long-term pair bonds.

All transmitters were solar powered neck collars and weighed <2% of the body mass (Bodey et al. 2018; Phillips et al. 2003). Tagging was performed under licence from the British Trust for Ornithology's Special Methods Technical Panel. For both species, data from GSM tags were downloaded remotely via the 3G cellular network, whilst data from Pathtrack and Ecotone GPS-UHF tags was transmitted via ultra-high frequency radio to base stations. For Ecotone and Ornitela tags GPS fixes were taken continuously whilst Pathtrack tags only collected data during the day. We removed fixes from the day of capture to minimise the influence of capture on behavior. We only retained data from individuals with at least 5 separate days of data during each winter and therefore obtained data from 33 GBG tags over two winters and 94 GWfG tags over eight winters.

GPS data were obtained from all tag types but triaxial accelerometer data was only obtained from Ecotone and Ornitela GSM tags. The sampling regimes for each tag type was as follows:

1. GBG Pathtrack UHF- 30 min GPS fixes during the daytime
2. GBG Ecotone GSM- 30 min GPS fixes during the daytime and 60 min GPS fixes during the night, 3.2 sec acceleration burst every 15 mins all day
3. GBG Ornitela GSM- 30 min GPS fixes during the daytime and 60 min GPS fixes during the night, 10 sec acceleration burst every 30-60 mins all day
4. GWfG Ecotone UHF- 60 min GPS fixes all day
5. GWfG Ecotone GSM- 60 min GPS fixes all day, 1.6 sec acceleration burst every 20 mins all day
6. GWfG Ornitela GSM- 15 min GPS fixes all day, 3 sec acceleration burst every 6 mins all day

For all tags the interval between fixes varied throughout the winter due to low battery recharge through the solar panel. Therefore, the sampling regimes above are the maximum sampling rates of each tag type and all GPS tracking data were re-sampled to 1 hour for all analysis.

## Section S2: Classification of accelerometer data into behaviors

To assess the effect of shooting disturbance on time-activity budgets of GWfG we used accelerometer data that had been classified, using a machine learning algorithm, into four distinct behaviors: walking, grazing, stationary and flying

We initially created a training data set that linked known observed behaviors to the accelerometer data recorded by the tag. This was achieved by periodically collecting continuous accelerometer data from 9 different Ornitela GPS-GSM tags and simultaneously recording individuals using a Canon SX70 HS video camera with 60x digital zoom. These birds could be individually recognised due to unique codes painted onto their neck collar tags. The accelerometer data was labelled with five unique observed behaviors: walking, grazing, alert, resting and flying. The continuous accelerometer data was then split up into 3sec 10HZ bursts, the same length and frequency collected by the tags for the rest of the year. If two consecutive bursts had the same behavior, one was removed to try and increase the independence between bursts. Flight bursts were recorded rarely during direct observation, so we supplemented the training data set with additional bursts during periods of active migration when birds were over the North Atlantic and travelling more than 10km/h to provide guaranteed flight bursts. This gave us the following number of bursts per behavior

in the training dataset: alert = 472; flying = 1753; grazing = 1689; resting = 287 and walking =376.

The training dataset was uploaded to the AccelerRator online application (Resheff et al. 2014), and several machine learning algorithms were trailed including, nearest neighbour, linear support vector machine (SVM), Radial basis function SVM, Random Forest and linear discriminant analysis. The models were tested by splitting the training data set 70:30, training the algorithm on the 70% and then testing the accuracy of classification on the remaining 30%. Radial basis function SVM and Random Forest performed identically across three measures of accuracy (sensitivity, specificity, and recall) (Appendix S1: Figure S1). Random forest was chosen due its popularity for this type of classification and ease of implementation in R (Nathan et al. 2012, Clermont et al. 2021). In addition, we chose to combine the alert and resting classes into a single category, stationary. These two categories had the lowest precisions and recall and were often misclassified as one another.

We then used the *caret* package (Kuhn 2021) to build our own random forest model as this enabled us to choose the values of two hyperparameters, *ntree* (number of trees) and *mtry* (the number of variables to randomly sample as candidates at each split). Larger *ntree* values increase accuracy but at a value of 5000 accuracy asymptotes, *mtry* helps to balance low correlation between trees with high predictive strength of each tree. We derived 49 different summary statistics from our tri-axial accelerometer data that were used by the random forest model (Appendix S1: Table S1). Our data set was split 70:30, using 70% for model training and 30% as a test set to assess the accuracy of our final random forest model. We *ntree*=5000 and tuned a single hyper-parameter, *mtry*, with 10-fold cross validation using the

'*train'* function in the *caret* (Kuhn 2021) package. We selected *mtry*=6 but any value between 3 and 11 resulted in only a 0.1% difference in classification accuracy. The accuracy of the final random forest model was 0.985 [95% CI: 0.977, 0.991] and the classification confusion matrix can be found in (Appendix S1: Table S2). Using this model, we classified all unclassified accelerometer data from GWfG on Islay with Ornitela GPS-GSM tags.

## References

Clermont, J., S. Woodward-Gagné, and D. Berteaux. 2021. Digging into the behaviour of an active hunting predator: arctic fox prey caching events revealed by accelerometry. Movement Ecology 9:1–12.

Kuhn, M. 2021. caret: Classification and Regression Training. R package version 6.0-90.

Nathan, R., O. Spiegel, S. Fortmann-Roe, R. Harel, M. Wikelski, and W. M. Getz. 2012. Using tri-axial acceleration data to identify behavioral modes of free-ranging animals: general concepts and tools illustrated for griffon vultures. Journal of Experimental Biology 215:986–996.

Resheff, Y. S., S. Rotics, R. Harel, O. Spiegel, and R. Nathan. 2014. AcceleRater: a web application for supervised learning of behavioral modes from acceleration measurements. Movement Ecology 2:1–7.

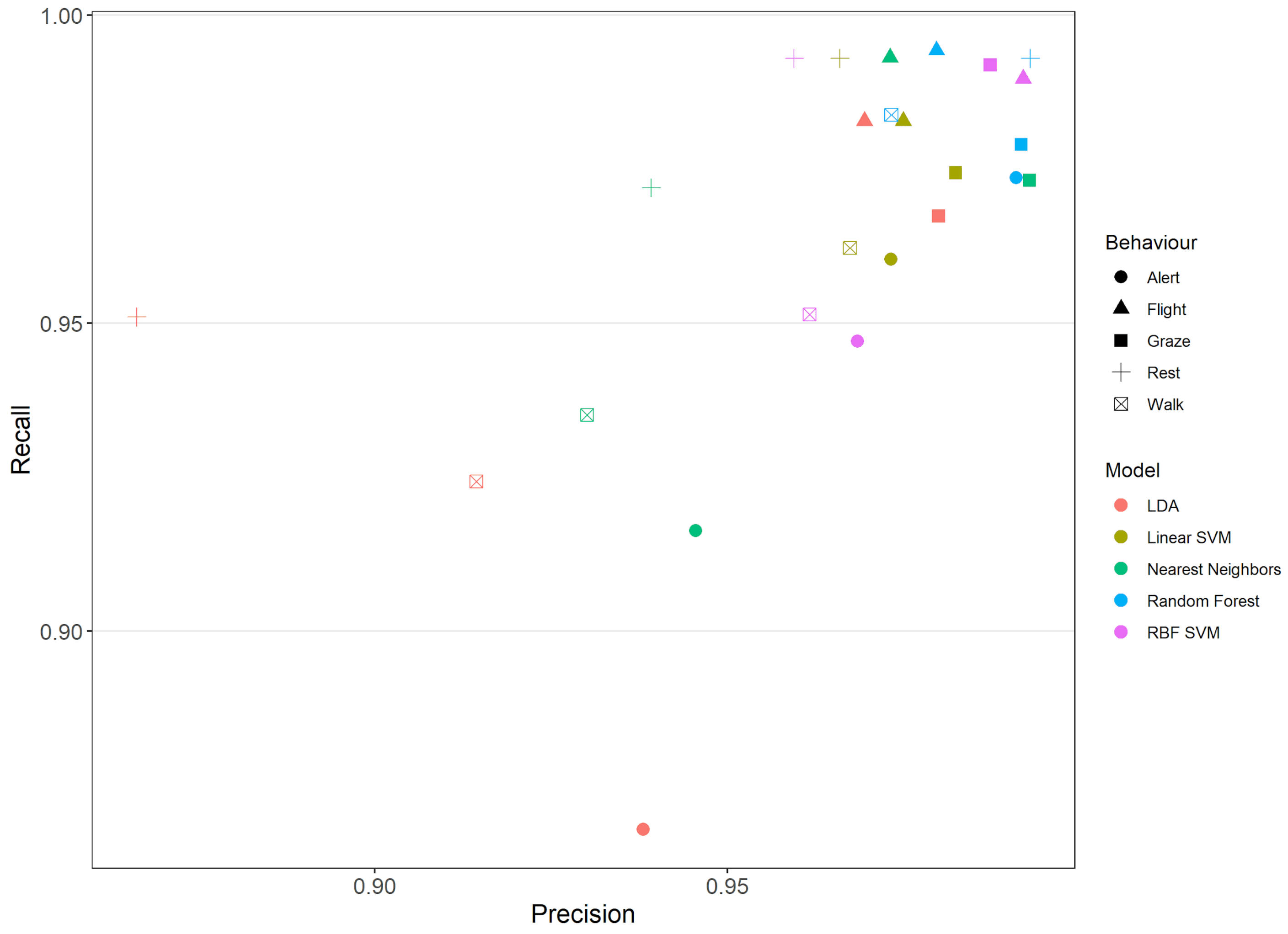


**Figure S1**. Precision vs Recall for the classification of accelerometer data into 5 different behaviors using five different machine learning algorithms. The AccelerRater online web application was used to run each of the machine learning algorithms.

**Table S1**. 49 summary statistics derived from triaxial accelerometer data recorded from GWfG. These were used as explanatories in a Random forest model that was used to classify the accelerometer data into ecologically meaningful behaviors.

| Name | Description | Axis for calculation |
|---|---|---|
| ODBA | Overall dynamic body acceleration: $ODBA = DBA_x + DBA_y + DBA_z$ | All |
| DBA | Dynamic body acceleration of each axis: $DBA = \frac{\sum \lvert(x - \underline{x})\rvert}{n}$ | Measured for each axis individually |
| Vector Norm | $Vector\ norm = \sqrt[2]{\sum x^2}$ | Measured for each axis individually |
| Min | Minimum of each axis | Measured for each axis individually |
| Max | Maximum of each axis | Measured for each axis individually |
| SD | Standard deviation of each axis | Measured for each axis individually |
| Skewness | Skewness of each axis | Measured for each axis individually |
| Kurtosis | Kurtosis of each axis | Measured for each axis individually |
| 25$^{th}$ Quantile | 25th Quantile of each axis | Measured for each axis individually |
| 50$^{th}$ Quantile | 50th Quantile of each axis | Measured for each axis individually |
| 75$^{th}$ Quantile | 75th Quantile of each axis | Measured for each axis individually |
| Mean difference | The mean difference between two axes | All combinations of two axis |
| SD of difference | Standard deviation of difference between two axes | All combinations of two axis |
| Covariance | Covariance between two axes | All combinations of two axis |
| Line crossings | Number of instances that two axes cross each other | All combinations of two axis |
| Wave amplitude | Mean local difference between minimum and maximum values, local values calculated in blocks of ten readings | Measured for each axis individually |

**Notes:** *ODBA*; overall dynamic body acceleration. *DBA*; dynamic body acceleration. *Min*: minimum. *Max*; maximum. *SD*; standard deviation.

**Table S2**. Confusion matrix for test data (30% of the training data) classifying four different behaviors (Flight, graze, stationary and walk) using out final random forest model. Values on the diagonal are true positive classification and those above and below the diagonal are false positive classifications.

| | | **Reference** | | | |
|---|---|---|---|---|---|
| | | **flight** | **graze** | **stationary** | **walk** |
| **Prediction** | **flight** | 520 | 7 | 0 | 0 |
| | **graze** | 5 | 499 | 0 | 0 |
| | **stationary** | 0 | 0 | 225 | 1 |
| | **walk** | 0 | 0 | 2 | 111 |
