# Supplementary material for: Contrasting effects of shooting disturbance on the movement and behavior of sympatric wildfowl species
Source: Ecol Appl. 2024 Oct 25;34(8):e3032. doi: 10.1002/eap.3032 (PMC11610659; doi:10.1002/eap.3032)
Supplement: Supplementary file 2 — Appendix S2. [file EAP-34-e3032-s001.pdf]

Appendix S2

Contrasting effects of shooting disturbance on the movement and behavior of sympatric wildfowl species

Luke Ozsanlav-Harris, Aimée L.S. McIntosh, Larry R. Griffin, Geoff M. Hilton, Lei Cao, Jessica M. Shaw & Stuart Bearhop

Journal: Ecological Applications

Table S1. Model selection table for generalised linear models for between-day comparisons of habitat use of GBG and GWfG on Islay according to daily shooting disturbance.

Species	*Coefficients*	Δ*AIC*	*AUC*	*k*	*Sensitivity*	*Specificity*	*Positive predictive power*	*Negative predictive power*	*Accuracy*
GBG	Habitat + Shot + Habitat*Shot	0	0.82	14	0.90	0.75	0.26	0.99	0.76
GBG	Habitat + Shot + Road	626.5	0.82	8	0.90	0.75	0.57	0.99	0.76
GWfG	Habitat + Shot + Habitat*Shot	0	0.82	14	0.81	0.82	0.32	0.98	0.82
GWfG	Habitat + Shot + Road	389.1	0.82	8	0.81	0.82	0.32	0.98	0.82

Notes: Models were ranked using AIC (Akaike information criterion) and run separately for each species to allow different pseudo absences to be created for each species. *k*; the number of parameters estimated in the model. *AUC*; area under the receiver operating curve (a measure of model fit). *Habitat*; fixed effect variable of seven habitat categories (seven-level factor). *Disturbance*; fixed effect variable indicating if birds were exposed to shooting on a given day (two-level factor).

Table S2. Estimates for the habitat*shooting interaction effect for the best performing resource selection models for between-day comparisons of shooting and non-shooting days (as a two-way interaction) and within-day comparisons of shooting days pre- and post-shooting disturbance (as a three-way interaction with distance to the nearest road). In both models the habitat class “other” acts as the reference class.

Model	Habitat Variable	Species	Estimate of shooting interaction
Between-day	Saltmarsh and Coastal	GBG	3.40 (0.32)
Between-day	Other grassland	GBG	3.58 (0.32)
Between-day	Improved grassland	GBG	3.21 (0.32)
Between-day	Freshwater	GBG	3.54 (0.40)
Between-day	Bog	GBG	3.94 (0.32)
Between-day	Arable	GBG	3.32 (0.32)
Between-day	Saltmarsh and Coastal	GWfG	0.70 (0.07)
Between-day	Other grassland	GWfG	0.44 (0.07)
Between-day	Improved grassland	GWfG	-0.02 (0.06)
Between-day	Freshwater	GWfG	-0.07 (0.11)
Between-day	Bog	GWfG	-0.14 (0.07)
Between-day	Arable	GWfG	0.34 (0.07)
Within-day	Saltmarsh and Coastal	GBG	-0.002 (0.24)
Within-day	Improved grassland	GBG	0.42 (0.18)
Within-day	Arable	GBG	1.56 (0.40)
Within-day	Other grassland	GWfG	2.24 (0.30)
Within-day	Improved grassland	GWfG	1.553(0.28)
Within-day	Arable	GWfG	-4.40 (0.57)

Table S3 Generalised linear models for habitat use of GBG on Islay according to the shooting disturbance status of an individual (pre- and post- shooting disturbance) in relation to distance from nearest road (m).

Coefficients	*ΔAIC*	*AUC*	*k*	*Sensitivity*	*Specificity*	*Positive predictive power*	*Negative predictive power*	*Accuracy*
Habitat + Shot + Road + Habitat* Shot *Road	0	0.8047	16	0.88934	0.72000	0.24106	0.98486	0.7354
Habitat + Shot + Road + Habitat* Shot + Habitat*Road + Shot *Road	13.5	0.8047	13	0.88852	0.72082	0.24143	0.98477	0.7367
Habitat + Shot + Road + Habitat*Road + Habitat* Shot	108.3	0.8062	10	0.89180	0.72057	0.24194	0.98521	0.7361
Habitat + Shot + Road + Habitat* Shot + Shot *Road	110.6	0.8013	12	0.88607	0.71656	0.23816	0.98435	0.732
Habitat + Shot + Road + Habitat*Road	118.3	0.8033	9	0.88852	0.71811	0.23966	0.98471	0.7336
Habitat + Shot + Road + Habitat* Shot + Shot *Road	259.7	0.8036	10	0.88934	0.71779	0.23962	0.98482	0.7334
Habitat + Shot + Road + Road* Shot	336.2	0.8043	7	0.89344	0.71525	0.23883	0.98532	0.7314
Habitat + Shot + Road + Habitat *Shot	341.1	0.7997	9	0.88689	0.71246	0.23573	0.98437	0.7283
Habitat + Shot + Road	410.8	0.8007	6	0.88852	0.71279	0.23627	0.98460	0.7288

Notes: Models were ranked using AIC and run separately for each species so that species-specific pseudo absences could be used. *k*; the number of parameters estimated in the model. *AUC*; area under the receiver operating curve (model fit measure). *Habitat*; fixed effect categorical variable (four-levels) of habitat types that differed between species. Habitats with less than 50 "real" fixes for each time period were grouped as "Other". *Disturbance*; fixed effect variable indicating period before (pre-) or after (post-) shooting disturbance on a shooting day (two-level factor). *Road*; fixed effect variable of distance to the nearest road (continuous).

Table S4 Generalised linear models for habitat use of GWfG on Islay according to the shooting disturbance status of an individual (pre- and post- shooting disturbance) in relation to distance from nearest road (m).

Coefficients	∆AIC	AUC	k	Sensitivity	Specificity	Positive predictive power	Negative predictive power	Accuracy
Habitat + Shot + Road + Habitat* Shot *Road	0	0.817	16	0.86087	0.77309	0.27504	0.98232	0.7811
Habitat + Shot + Road + Habitat* Shot + Habitat*Road + Shot *Road	205.8	0.8159	13	0.85652	0.77522	0.27591	0.98183	0.7826
Habitat + Shot + Road + Habitat* Shot + Shot *Road	245.5	0.8166	12	0.86135	0.77193	0.27414	0.98236	0.7801
Habitat + Shot + Road + Habitat*Road + Habitat* Shot	381.5	0.81112	10	0.85459	0.76787	0.26909	0.98142	0.7758
Habitat + Shot + Road + Habitat* Shot + Shot *Road	403.4	0.8156	10	0.84879	0.78242	0.28063	0.98104	0.7884
Habitat + Shot + Road + Habitat*Road	421.9	0.8123	9	0.85845	0.76623	0.26859	0.98186	0.7746
Habitat + Shot + Road + Habitat *Shot	469.9	0.8131	9	0.84155	0.78464	0.28097	0.98021	0.7898
Habitat + Shot + Road + Road* Shot	608.9	0.8103	7	0.82899	0.79155	0.28453	0.97885	0.7949
Habitat + Shot + Road	661.7	0.8088	6	0.82174	0.79594	0.28709	0.97809	0.7983

Notes: Models were ranked using AIC and run separately for each species so that species-specific pseudo absences could be used. *k*; the number of parameters estimated in the model. *AUC*; area under the receiver operating curve (model fit measure). *Habitat*; fixed effect categorical variable (four-levels) of habitat types that differed between species. Habitats with less than 50 “real” fixes for each time period were grouped as “Other”. *Disturbance*; fixed effect variable indicating period before (pre-) or after (post-) shooting disturbance on a shooting day (two-level factor). *Road*; fixed effect variable of distance to the nearest road (continuous).

Table S5 Model selection table to understand the effect of shooting disturbance on average daily ODBA, a proxy for energy expenditure. Sex, number of accelerometer bursts recorded each day *(N)* and days since November 1st as a quadratic term *(Date^2)* were included in all models as a fixed effects and individual ID and winter as a random intercept term. For each variable the parameter estimates and 95% confidence intervals are given if it is included in a given model.

N	***Sex***	***Date***	***Date^2***	***Shot***	***Sp***	***Shot*Sp***	***k***	***AIC***	**Δ*AIC***
0.021 [0.020,0.021]	-0.015 [-0.154,0.125]	2.937 [0.044,5.830]	-2.973 [-5.052,-0.893]	-	-	-	12	33038.61	0
0.021 [0.020,0.022]	-0.078 [-0.262,0.105]	2.812 [-0.912,5.715]	-3.050 [-5.133,-0.967]	-	-0.088 [-0.257,0.080]	-	13	33039.30	0.93
0.021 [0.020,0.021]	-0.014 [-0.153,0.125]	2.924 [0.030,5.817]	-2.980 [-5.060,-0.900]	-0.008 [-0.042,0.027]	-	-	15	33040.18	1.81
0.021 [0.020,0.022]	-0.078 [-0.261,0.106]	2.801 [-0.102,5.704]	-3.058 [-5.142,-0.974]	-0.008 [-0.042,0.027]	-0.089 [-0.257,0.080]	-	13	33041.11	2.74
0.021 [0.020,0.022]	-0.078 [-0.262,0.105]	2.806 [-0.099,5.710]	-3.060 [-5.144,-0.976]	0.017 [-0.060,0.094]	-0.088 [-0.257,0.080]	-0.030 [-0.116,0.055]	14	33042.62	4.25

Notes: Models were ranked using AIC. *k*; the number of parameters estimated in the model. *ODBA*; Overall Dynamic body acceleration. *Shot*; fixed effect variable indicating if birds were exposed to shooting on a given day or not (two-level factor). *Sp*; fixed effect variable indicating the two species GWfG and GBG (two-level factor).

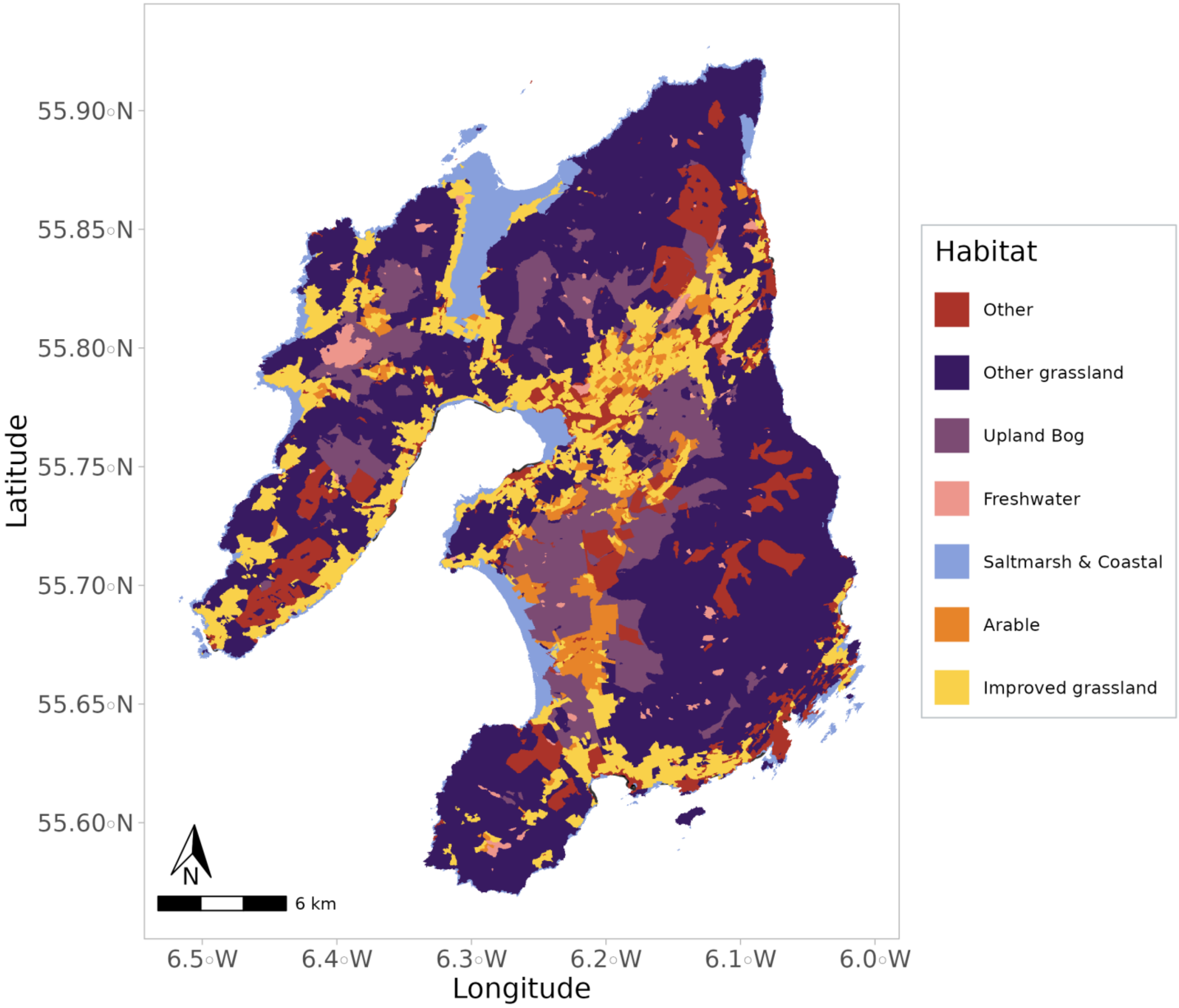

Figure S1 Habitat map of Islay, Scotland in 2018. Habitat classification was derived from a 20mx20m resolution landcover map of Britain (Morten et al. 2020). Some habitat categories from Morten et al. (2020) were grouped to aid habitat selection analysis model interpretation.

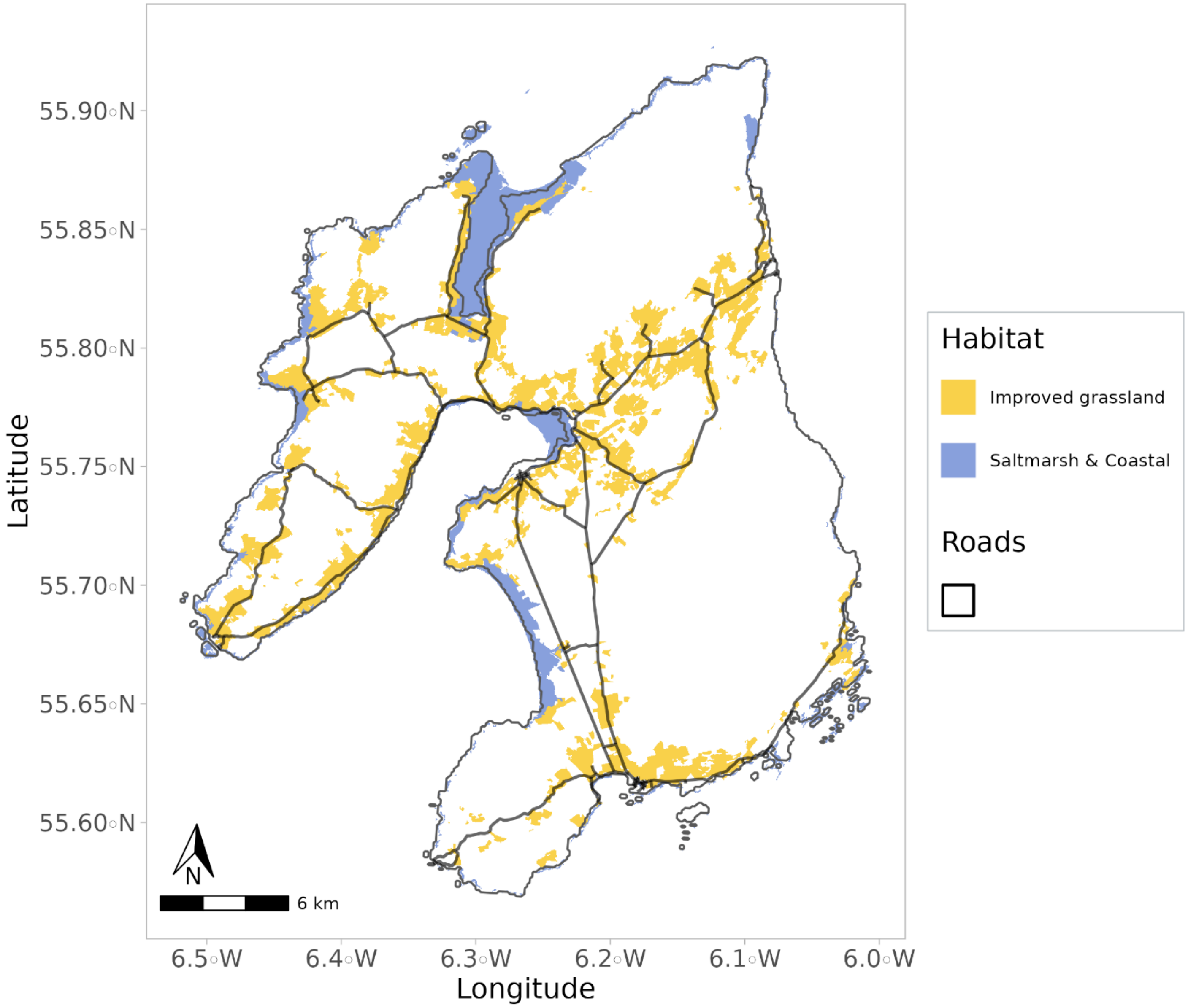

Figure S2 Map of Islay depicting the location of roads as well as the `Improved grassland` and `Saltmarsh and Coastal' habitats. Habitat classification was derived from a 20mx20m resolution landcover map of Britain (Morten et al. 2020).

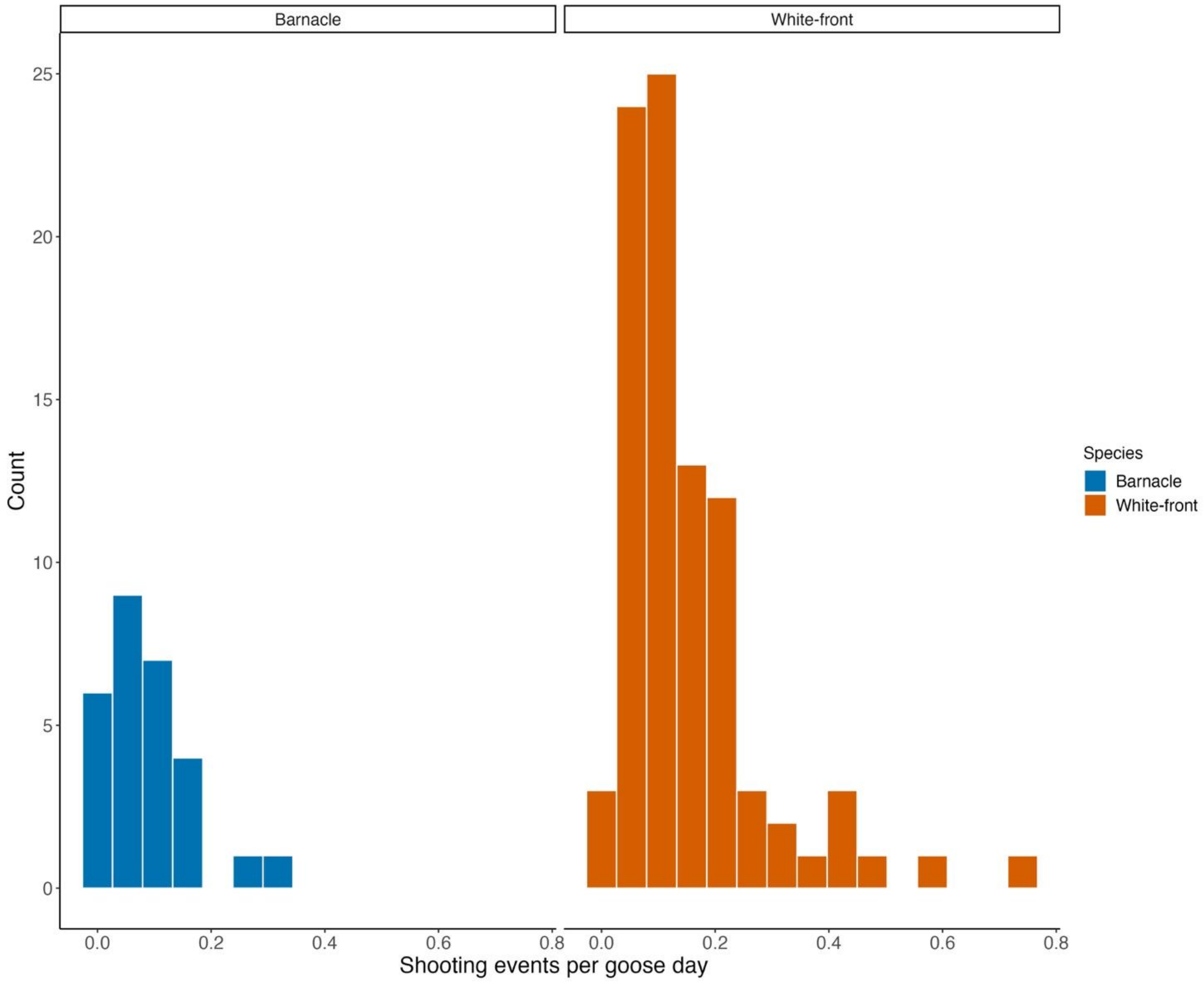

Figure S3. The rate of shooting disturbance per individual goose calculated as the total number of disturbance events an individual experiences per goose-day within a winter. A goose-day is a single day for one individually tagged bird within a winter. 0.3 shooting events per goose-day equates to 45 disturbance events over the course of an entire winter, 0.2 to 30 disturbance events and 0.1 to 15 disturbance events.

References

Morton, R. D., C. G. Marston, A. W. O'Neil, and C.S. Rowland. 2020. "Land Cover Map 2019 (20m Classified Pixels); GB_ NERC Environmental Information Data Centre."
